# Primer choice shapes microbial community interpretation across habitats and informs short-term structured enrichment in environmental and applied systems

**DOI:** 10.3389/fmicb.2026.1838890

**Published:** 2026-05-29

**Authors:** Marta Velaz Martín, Hagen Rießland, Kersten S. Rabe, Christof M. Niemeyer

**Affiliations:** Karlsruhe Institute of Technology (KIT), Institute for Biological Interfaces 1 (IBG-1), Biomolecular Micro- and Nanostructures, Eggenstein-Leopoldshafen, Germany

**Keywords:** amplicon sequencing, environmental monitoring, metagenomics, microbial community profiling, microbial enrichment, primer bias, soil microbiome

## Abstract

Microbial communities play central roles in ecosystem functioning across natural and engineered environments, yet their accurate characterization remains challenging due to methodological biases in amplicon sequencing. Primer choice can strongly influence taxonomic resolution, diversity estimates, and ecological interpretation. Here, we systematically compared primer performance across multiple ribosomal marker genes (16S, 18S, 28S rRNA, and ITS) and contrasting habitats, including soil, wastewater, and a photobioreactor-derived suspension. Amplicon-based profiles were benchmarked against shotgun metagenomic data. Primer choice significantly affected community composition, diversity metrics, and concordance with metagenomic profiles across all habitats and markers. Although 16S rRNA gene primers targeting the V3 region showed the highest agreement, no primer set fully reconstructed community structure. Applying the best-performing primer to a structured soil enrichment system using MESIF chips revealed rapid divergence from native soil and convergence toward less diverse communities, consistently favoring copiotrophic, surface-associated taxa while characteristic soil taxa declined. Across the 21-day incubation period, MESIF-associated communities diverged strongly from native soil, whereas medium-specific differences were comparatively smaller. This suggests that early enrichment was dominated by colonization of the structured matrix, while longer incubations and functional analyses will be needed to resolve substrate-specific selection. Overall, our findings highlight primer selection as a critical factor in microbial community analysis and show that combining optimized amplicon sequencing with structured cultivation enables reproducible enrichment, improved community monitoring, and targeted recovery of functionally relevant microorganisms. These insights are relevant for environmental monitoring, wastewater treatment, biotechnology, and controlled environment agriculture.

## 1 Introduction

Microorganisms are the most abundant and diverse forms of life on Earth and play essential roles in ecosystem functioning (Flemming and Wuertz, 2019). In soils, microbial communities regulate nutrient cycling, organic matter turnover, and a wide range of biogeochemical processes that contribute to terrestrial ecosystem stability (Fierer, 2017; Al-Kaisi et al., 2017; Kuzyakov and Blagodatskaya, 2015). Complex microbial assemblages also drive key functions in engineered systems, including wastewater treatment and agricultural systems such as controlled environment agriculture (CEA) platforms (Glockow et al., 2024). In wastewater treatment plants, microbial consortia mediate nitrogen removal, carbon degradation, and contaminant transformation, while suspension communities in photobioreactors contribute to nutrient recycling and biomass production (Zhang et al., 2018; Yan et al., 2025; Glockow et al., 2023). Across these natural and engineered environments, microbial communities are highly diverse and dynamic, making accurate community profiling both essential and challenging.

Amplicon sequencing remains one of the most widely used approaches for microbial community analysis because it is high-throughput, scalable, and cost-effective (Liu et al., 2022; Lewis et al., 2021; Liu et al., 2021). However, PCR-based profiling is inherently affected by methodological bias (Klindworth et al., 2012; Parada et al., 2016; Thijs et al., 2017; Shaffer et al., 2025; Deissová et al., 2023; Barak et al., 2023; Beckers et al., 2016; Kounosu et al., 2019; Zheng et al., 2022). Different primer sets target different variable regions of ribosomal marker genes and can therefore influence taxon recovery, relative abundance estimates, and diversity patterns (Rathod and Silverman, 2026).

Primer bias can arise when primer–template mismatches reduce amplification efficiency or exclude specific taxa, and may also be influenced by amplicon length, GC content, taxonomic resolution, and uneven reference database coverage (Klindworth et al., 2012; Parada et al., 2016; Shaffer et al., 2025). These effects are particularly relevant in complex environmental samples, where closely related organisms may differ in primer binding sites and where many taxa remain poorly represented in curated databases. Consequently, primer choice can influence not only which taxa are detected, but also relative abundance patterns, diversity estimates, and downstream ecological interpretation (Thijs et al., 2017; Shaffer et al., 2025; Rathod and Silverman, 2026).

Although primer-dependent effects are well recognized, systematic comparisons across multiple habitats and ribosomal markers remain limited. In particular, amplicon-derived taxonomic profiles are still only occasionally compared with metagenome-derived reference profiles (Poretsky et al., 2014; Ranjan et al., 2016; Clooney et al., 2016). Such comparisons can help identify primer sets that most consistently represent the target marker community under defined experimental conditions. However, they must be interpreted carefully. Metagenome-derived ribosomal marker profiles provide a marker-matched reference generated without targeted marker-gene PCR amplification, but they do not represent a complete whole-metagenome reconstruction of community composition. Therefore, primer benchmarking should be framed as an assessment of agreement between targeted amplicon profiles and metagenome-derived reference profiles, rather than as validation against an absolute ground truth.

Most studies focus on a single marker and a single habitat, typically targeting either prokaryotic or eukaryotic communities, whereas few assess how primer bias differs between these domains or propagates into applied experimental systems. It therefore remains unclear how primer performance shapes downstream ecological conclusions, particularly when amplicon sequencing is used to interpret selection processes in enrichment experiments.

This issue is particularly relevant for cultivation-based enrichment systems, which are widely used to investigate microbial selection, explore functional potential, and recover previously uncultivated taxa, including members of the so-called microbial dark matter (Lewis et al., 2021; Lock, 2015; Zoheir et al., 2022; Zha et al., 2022; Samuel et al., 2025). Structured matrices such as bioinert macroporous elastomeric silicone foam (MESIF) chips mimic aspects of natural spatial heterogeneity and promote surface colonization and biofilm formation, both of which can strongly influence enrichment outcomes (Zoheir et al., 2022; Itani et al., 2026).

In these systems, observed community shifts may reflect a combination of biological selection and methodological detection bias. If a primer set underrepresents particular taxa, enrichment trajectories may be misinterpreted as ecological patterns rather than analytical artefacts. Conversely, benchmarking primer performance prior to applying an amplicon workflow can improve confidence that observed changes reflect reproducible community dynamics within the resolution limits of the selected marker.

**Figure 1:**
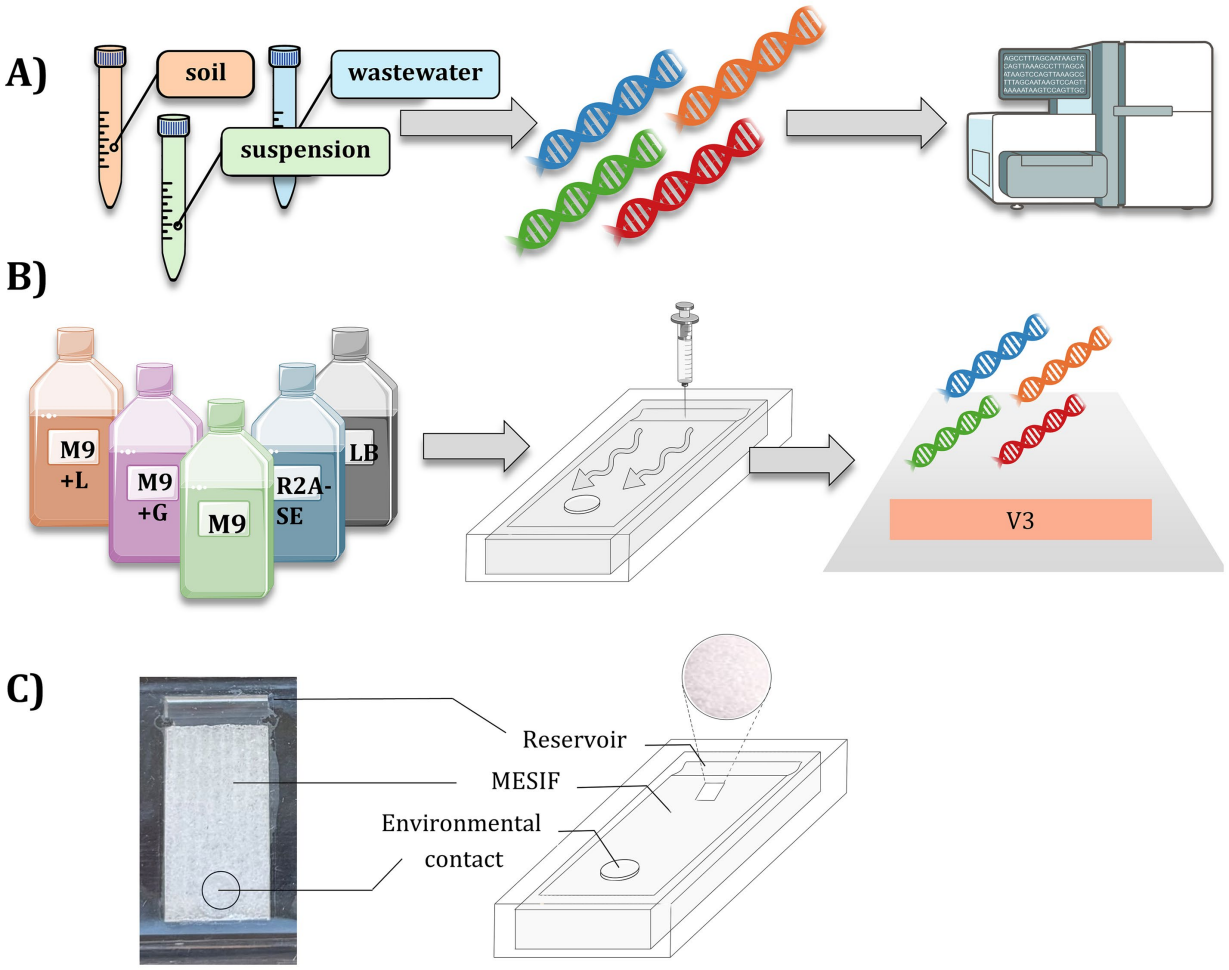
Overview of the study design integrating primer benchmarking and MESIF-based enrichment. **(A)** Environmental samples from soil, wastewater, and a photobioreactor-derived suspension were analyzed using amplicon sequencing with multiple primer sets targeting different ribosomal marker genes (16S, 18S, 28S rRNA, and ITS). Amplicon-based profiles were systematically compared to shotgun metagenomic data to assess primer-dependent biases in community composition, diversity, and taxonomic resolution. **(B)** Workflow of the MESIF-based enrichment system. Based on benchmarking results, the best-performing bacterial primer set (16S rRNA gene, V3 region) was selected and applied to characterize microbial communities during enrichment. MESIF chips loaded with different cultivation media were incubated in soil for 21 days, enabling microbial colonization under structured conditions. Following incubation, communities from MESIF chips and corresponding soil controls were analyzed to assess enrichment dynamics and community shifts. **(C)** Schematic representation of the MESIF chip design. The system consists of a macroporous matrix (MESIF), a reservoir enabling nutrient supply, and an environmental interface that facilitates microbial colonization from the surrounding soil.

Here, we systematically compare primer performance across multiple ribosomal markers and habitats and benchmark amplicon-derived taxonomic profiles against shotgun metagenomic data (Figures 1A,B). Based on this multi-criteria evaluation, we identify the best-performing bacterial primer set under the tested conditions and apply it to a structured soil cultivation system using MESIF chips supplemented with different media (Figure 1C). By combining methodological benchmarking with an applied enrichment workflow, we assess how primer choice influences ecological interpretation and how structured cultivation reshapes soil-derived microbial communities.

## 2 Materials and methods

### 2.1 Sample collection and environmental cultivation

#### 2.1.1 Environmental samples for primer benchmarking

To compare primer performance, three distinct environmental sample types representing contrasting microbial habitats were selected: soil, wastewater, and a photobioreactor-derived suspension. Soil samples were collected at KIT Campus North (49.096683, 8.432755). Wastewater samples were obtained from the chemical tank WP06 B01.1 of the KIT wastewater treatment plant (49.100337, 8.428108) (Zoheir et al., 2022). Suspension samples originated from an industrial-scale photobioreactor system operated by Acheron GmbH (Bremen, Germany), as previously described (Glockow et al., 2023). Each habitat was processed in three biological replicates (*n* = 3).

These environments were selected to capture a broad range of microbial community structures and ecological contexts. Soil represents a highly complex and taxonomically diverse terrestrial habitat, wastewater systems are characterized by dense and functionally specialized microbial consortia, and photobioreactor suspensions provide a controlled engineered environment with distinct community dynamics. Together, these sample types enabled the evaluation of primer performance across both natural and engineered microbial ecosystems. Amplicon and shotgun metagenomic sequencing were performed on the same DNA extracts to ensure direct comparability between methods.

#### 2.1.2 Macroporous elastomeric silicone foams for enrichment in soil

For cultivation-based enrichment experiments in soil, MESIF chips were employed (Supplementary Figure S1). The matrices were prepared as previously described (Zoheir et al., 2022). In brief, polydimethylsiloxane (PDMS; SYLGARD® 184, Germany) was mixed with curing agent (10:1, w/w), degassed, combined with sieved salt crystals (500–707 μm) as porogens, and cast into custom PMMA molds. Following centrifugation and thermal curing (70 °C), the solidified PDMS–salt composites were removed, extensively washed in warm water to leach out the salt, and dried, yielding the final porous cultivation matrices.

PDMS housings, consisting of cages and lids, were fabricated separately using PMMA molds as previously described (Zoheir et al., 2022). After thermal curing, an environmental contact hole was punched into the lid, which was then used as the environmental contact side of the MESIF chip. The assembled chip thus comprised a porous PDMS matrix enclosed within the housing, with the perforated lid enabling contact with the surrounding soil and an integrated reservoir chamber for medium loading. All components were treated with oxygen plasma to activate the surfaces for bonding and were assembled into complete MESIF chips under thermal curing and mechanical pressure. Each chip was subsequently loaded with 1 mL of cultivation medium via the reservoir chamber.

The tested media included Luria–Bertani broth (LB), M9 minimal medium without an additional carbon source (M9), M9 supplemented with 0.4% (w/v) glucose (M9 + G), M9 supplemented with 0.4% (w/v) lactose (M9 + L), and Reasoner’s 2A medium supplemented with soil extract (R2A-SE) (Chaudhary et al., 2019; Table 1). R2A-SE has previously been reported to support the cultivation of microbial dark matter (MDM) from soil environments.

**Table 1:** Cultivation media used for soil-based MESIF chip enrichment experiments.

Name	Composition
Luria-Bertani (LB)	5 g/L yeast extract, 20 g/L tryptone, 0.584 g/L NaCl, 0.186 g/L KCl, 2.4 g/L MgSO₄, 4 g/L glucose; pH 7.5
M9 Mineral	M9 salts* (1×), 4 g/L glucose, 1 mM MgSO₄, 0.3 mM CaCl₂, 1 mg/L biotin, 1 mg/L thiamine, trace elements** (1×)
	*M9 salt solution (10x): 75.2 g/L Na₂HPO₄·2H₂O, 30 g/L KH₂PO₄, 5 g/L NaCl, 5 g/L NH₄Cl **Trace elements solution (100x): 5 g/L EDTA, 0.83 g/L FeCl_3_-6H_2_ O, 84 mg/L ZnCl_2_, 13 mg/L CuCl_2_-2H_2_O, 10 mg/L CoCl_2_-2H_2_O, 10 mg/L H_3_BO_3_, 1.6 mg/L MnCl_2_-4H_2_ O
R2A-SE	0.5 g/L casein, 0.5 g/L glucose, 0.5 g/L peptone, 0.5 g/L sodium lactate, 0.5 g/L soluble starch, 0.5 g/L yeast extract, 0.05 g/L MgSO₄·7H₂O, 0.3 g/L K₂HPO₄, soil extract* *Soil extract (SE): prepared by suspending 1 kg soil in 2 L ddH_2_O, shaken overnight, centrifuged (3,400 rpm, 10 min), and sterile filtrated (0.2 μm)

The inoculated chips were incubated in soil for up to 21 days. Chips were placed horizontally in a pre-formed soil trench (4 cm depth) and loosely covered with soil (Supplementary Figure S2). Samples were collected at days 1, 3, 4, 7, 14, and 21 post-inoculation. For each medium and sampling time point, three independent MESIF chips were retrieved and processed as biological replicates (*n* = 3 per medium and time point). Native soil samples were collected in parallel and served as habitat controls. Following retrieval, the MESIF matrices were processed for DNA extraction and subsequent amplification of the 16S rRNA gene (V3 region; see Figure 1). All samples were stored at −20 °C immediately after collection until DNA extraction.

### 2.2 DNA extraction

Genomic DNA (gDNA) was extracted from liquid samples by centrifugation of 2 mL at 10,000 × g for 10 min. The resulting pellet was resuspended in CD1 lysis buffer (DNeasy PowerSoil Kit, QIAGEN, Germany) and processed according to the manufacturer’s instructions. For soil samples, DNA was extracted from 100 mg of starting material using the same kit.

For MESIF samples, the porous silicone matrix was removed from its housing and manually compressed in CD1 lysis buffer for 1 min to release retained biomass prior to extraction following the standard protocol.

DNA concentration and purity were determined using fluorometric quantification (Qubit 3, Thermo Scientific Inc.) and spectrophotometric measurements (NanoDrop OneC, Thermo Scientific Inc.). All samples were processed using identical extraction procedures to ensure comparability across sample types.

### 2.3 Amplicon and shotgun sequencing

Each environmental sample (soil, wastewater, and suspension) was analyzed using both amplicon sequencing and shotgun metagenomics to enable direct comparison between targeted marker-gene approaches and whole-community genomic profiles. Amplicon and shotgun datasets were generated from the same DNA extracts to ensure comparability between methods.

Amplicon sequencing targeted multiple ribosomal marker regions, including the prokaryotic 16S rRNA gene and the eukaryotic 18S and 28S rRNA genes, as well as the internal transcribed spacer (ITS) region, to capture taxonomic diversity across domains. Primer sets were selected based on previously published studies and their reported performance across diverse environments (Table 2).

**Table 2:** Primer sets used in this study, including target genes, primer sequences, amplified regions and targeted microbial domains.

Target gene	Name	Forward primer (5′–3′)	Reverse primer (5′–3′)	Amplified region	Target domain
16S rRNA	16S-V3	CCTACGGGNGGCWGCAG	WTTACCGCRGCTGCTGG	V3 (Muyzer et al., 1993)	Prokaryota
16S rRNA	16S-V4	GTGCCAGCMGCCGCGGTAA	GGACTACHVGGGTWTCTAAT	V4 (Deissová et al., 2023)	Prokaryota
16S rRNA	16S-V2-V3	AGAGTTTGATCMTGGCTCAG	TGCTGCCTCCCGTAGGAGT	V2–V3 (Barak et al., 2023)	Prokaryota
16S rRNA	16S-V6-V7	AACMGGATTAGATACCCKG	ACGTCATCCCCACCTTCC	V6–V7 (Beckers et al., 2016)	Prokaryota
18S rRNA	18S-V9	GTACACACCGCCCGTC	TGATCCTTCTGCAGGTTCACCTAC	V9 (Kounosu et al., 2019)	Eukaryota
18S rRNA	18S-V9-2	CCCTGCCHTTTGTACACAC	CCTTCYGCAGGTTCACCTAC	V9 (Zheng et al., 2022)	Eukaryota
18S rRNA	18S-V3-4	GCGGTAATTCCAGCTCCAA	AATCCRAGAATTTCACCTCT	V3–V4 (Zheng et al., 2022)	Eukaryota
28S rRNA	28S-D3-D4	TTGAAACACGGACCAAGGAG	TTCGATTRGTCTTTCGCCCCT	D3–D4 (Kounosu et al., 2019)	Eukaryota
28S rRNA	28S-D4-D5	AGGGGCGAAAGACYAATCGAA	CRCCAGTTCTGCTTACCAAAA	D4–D5 (Kounosu et al., 2019)	Eukaryota
ITS	ITS-SSU	GGCTTGGTCATTTAGAGGAAGTAA	CGGCTGCGTTCTTCATCGATGC	SSU–5.8S (Op De Beeck et al., 2014)	Eukaryota (Fungi)
ITS	ITS-LSU	GCATCGATGAAGAACGCAGC	TCCTCCGCTTATTGATATGC	5.8S–LSU (Op De Beeck et al., 2014)	Eukaryota (Fungi)
ITS	ITS-SSU-LSU	GTCGTAACAAGGTAGCCGTA	GCCAAGGCATCCACC	SSU–LSU (Cardinale et al., 2004)	Eukaryota

In parallel, shotgun metagenomic sequencing was performed on the same DNA extracts to provide an amplification-independent taxonomic reference. Shotgun libraries were generated from purified genomic DNA without prior target enrichment.

Primer performance was evaluated based on alpha diversity, taxonomic composition, and concordance with metagenome-derived ribosomal marker profiles.

The primer set showing the most consistent performance across these criteria (16S-V3) was selected for downstream analysis (see Section 2.4). Samples from the MESIF enrichment experiment were subsequently analyzed only by 16S rRNA gene amplicon sequencing using the selected primer set (16S-V3).

#### 2.3.1 PCR amplification

Amplicon PCRs were performed using marker-specific primer pairs (Table 2) incorporating Illumina adapter overhangs (forward: ACACTCTTTCCCTACACGACGCTCTTCCGATCT; reverse: GACTGGAGTTCAGACGTGTGCTCTTCCGATCT). Reactions were prepared in a final volume of 25 μL containing Q5 High-Fidelity DNA Polymerase (New England BioLabs), 1 × Q5 reaction buffer, High GC enhancer, 0.25 μM of each primer, 0.2 mM dNTPs, and 20 ng of template DNA.

PCR amplification was performed using a touchdown PCR protocol. An initial denaturation step at 95 °C for 2 min was followed by four touchdown cycles consisting of denaturation at 95 °C for 40 s, annealing for 40 s with stepwise temperature reduction and elongation at 72 °C for 40 s. Annealing temperatures were decreased from 56–52 °C for 16S rRNA primers, 60–56 °C for 18S rRNA and ITS primers, and 64–60 °C for 28S rRNA primers. This was followed by 20 amplification cycles with denaturation at 95 °C for 40 s, annealing at the final primer-specific temperature (52 °C for 16S rRNA primers, 56 °C for 18S rRNA and ITS primers, and 60 °C for 28S rRNA primers) for 40 s, and elongation at 72 °C for 40 s. A final elongation step at 72 °C for 5 min completed the amplification. All PCR reactions were performed in technical duplicates. Technical duplicate PCRs were processed independently during library preparation and sequenced separately, rather than being pooled prior to sequencing. Negative controls without template DNA were included during PCR amplification to monitor amplification-related contamination. These controls did not yield amplicon material suitable for library preparation and were therefore not sequenced.

PCR products were purified using the Clean & Concentrate-5 Kit (Zymo Research, Germany) according to the manufacturer’s instructions.

#### 2.3.2 Library preparation and sequencing

Sequencing libraries were prepared from both purified genomic DNA (for shotgun metagenomics) and purified amplicon PCR products using the NEBNext Ultra II FS DNA Library Prep Kit (New England BioLabs, Germany), following the manufacturer’s protocol for DNA inputs ≥100 ng. For shotgun libraries, enzymatic fragmentation was performed for 14 min, followed by adapter ligation and indexing using six PCR cycles. Amplicon libraries were prepared without additional fragmentation.

Library size distribution and quality were assessed using the Agilent High Sensitivity DNA Kit on an Agilent 2,100 Bioanalyzer (Agilent Technologies, Germany). Paired-end sequencing was conducted on an Illumina NextSeq1000 platform using a High Output Kit v2.5 (300 cycles).

### 2.4 Bioinformatic analysis

#### 2.4.1 Marker-gene based analysis

Amplicon datasets from all ribosomal markers (16S, 18S, and 28S rRNA genes, as well as the ITS region) were processed using QIIME 2 (version 2021.8.0) (Bolyen et al., 2019). Raw paired-end reads were quality filtered and adapter-trimmed using fastp (Chen et al., 2018), and subsequently merged using FLASH (Magoč and Salzberg, 2011) to generate full-length amplicon sequences. Merged reads were imported into QIIME 2, and residual primer sequences were removed using cutadapt (Martin, 2011).

Amplicon sequence variants (ASVs) were inferred using DADA2 (Callahan et al., 2016), which includes error correction and dereplication. Chimeric sequences were identified and removed using VSEARCH (Rognes et al., 2016). ASV sequences were aligned using MAFFT via the q2-alignment plugin, and phylogenetic trees were constructed using FastTree2 via the q2-phylogeny plugin where applicable (Katoh and Standley, 2013). For diversity analyses, feature tables were rarefied within each marker to control for differences in sequencing depth.

Taxonomic classification of ASVs was performed using marker-specific reference databases within QIIME 2. For 16S and 18S rRNA gene amplicons, taxonomic assignment was carried out using Naïve Bayes classifiers trained on the SILVA reference database (release 138.2) (Quast et al., 2013). ITS amplicons were classified using the UNITE dynamic developer release (version 10.0), while 28S rRNA gene amplicons were classified using the SILVA LSU database (release 138.2) (Abarenkov et al., 2023).

#### 2.4.2 Shotgun metagenome analysis

Shotgun metagenomic data were processed to generate PCR-independent ribosomal marker profiles for comparison with amplicon-derived taxonomic profiles. This approach was selected to provide a marker-based reference derived from the same DNA extracts, independent of PCR amplification. Raw paired-end reads were quality filtered and adapter-trimmed using fastp (Chen et al., 2018). Overlapping read pairs were merged using FLASH to improve downstream marker recovery and assembly (Magoč and Salzberg, 2011).

Preprocessed reads were assembled using MEGAHIT (Li et al., 2015). Ribosomal marker sequences from the small subunit (SSU) and large subunit (LSU) were extracted from the assemblies using the get_markers function within MDMCleaner (Vollmers et al., 2022). To quantify marker abundances, reads were mapped back to the extracted marker sequences using CoverM (Samuel et al., 2025), and relative abundances were calculated based on read coverage. Taxonomic profiles were visualized using KRONA (Ondov et al., 2013).

We emphasize that this workflow does not represent a whole-metagenome shotgun taxonomic profiling approach based on genome-wide information. Instead, it provides a metagenome-derived ribosomal marker reference that is directly comparable to the targeted amplicon datasets.

#### 2.4.3 Data integration and statistical analysis

Downstream data integration, statistical analyses, and visualization were performed in R (version 4.5.1) using phyloseq (McMurdie and Holmes, 2013). Feature tables, taxonomic assignments, and sample metadata generated in QIIME 2 were imported into R and combined into phyloseq objects for each marker.

For quality control and diversity analyses, sequencing depth was summarized per sample and primer set before rarefaction. For diversity analyses, observed ASV counts and Shannon diversity, were calculated from rarefied feature tables within each marker to control for differences in sequencing depth and summarized by habitat and primer. Shannon values correspond to the standard Shannon diversity index and were not transformed to effective numbers of ASVs. Beta diversity was assessed using Bray–Curtis dissimilarities on relative abundance data and Jaccard distances on presence–absence data. Ordination was performed using non-metric multidimensional scaling (NMDS).

To quantify enrichment dynamics, Bray–Curtis distances between MESIF samples and their corresponding native soil communities (“distance to soil”) were calculated across time points. To assess primer-specific detection patterns, the most and least abundant families were compared across primer sets using a dot plot approach, stratified by habitat and domain. Concordance between amplicon and metagenomic profiles was evaluated at the family level using Spearman rank correlation (Schober et al., 2018).

Differential abundance analyses were performed using ANCOM-BC to identify taxa significantly enriched or depleted in MESIF samples relative to native soil controls. Analyses were conducted separately for each medium and time point. Multiple testing correction was performed using the Benjamini–Hochberg false discovery rate (FDR) procedure, and taxa with FDR-adjusted *p*-values < 0.05 were considered significantly differentially abundant.

### 2.5 Data availability

Amplicon sequences and metagenomic reads are publicly available in NCBI SRA under BioProject ID PRJNA1442665.

## 3 Results

### 3.1 Primer choice systematically reshaped community structure across habitats

#### 3.1.1 Primer-dependent differences in diversity

To assess the impact of primer choice across habitats, we analyzed three contrasting environments: soil, wastewater, and a photobioreactor-derived suspension. We first evaluated the effect of primer selection on within- and between-sample diversity.

**Figure 2:**
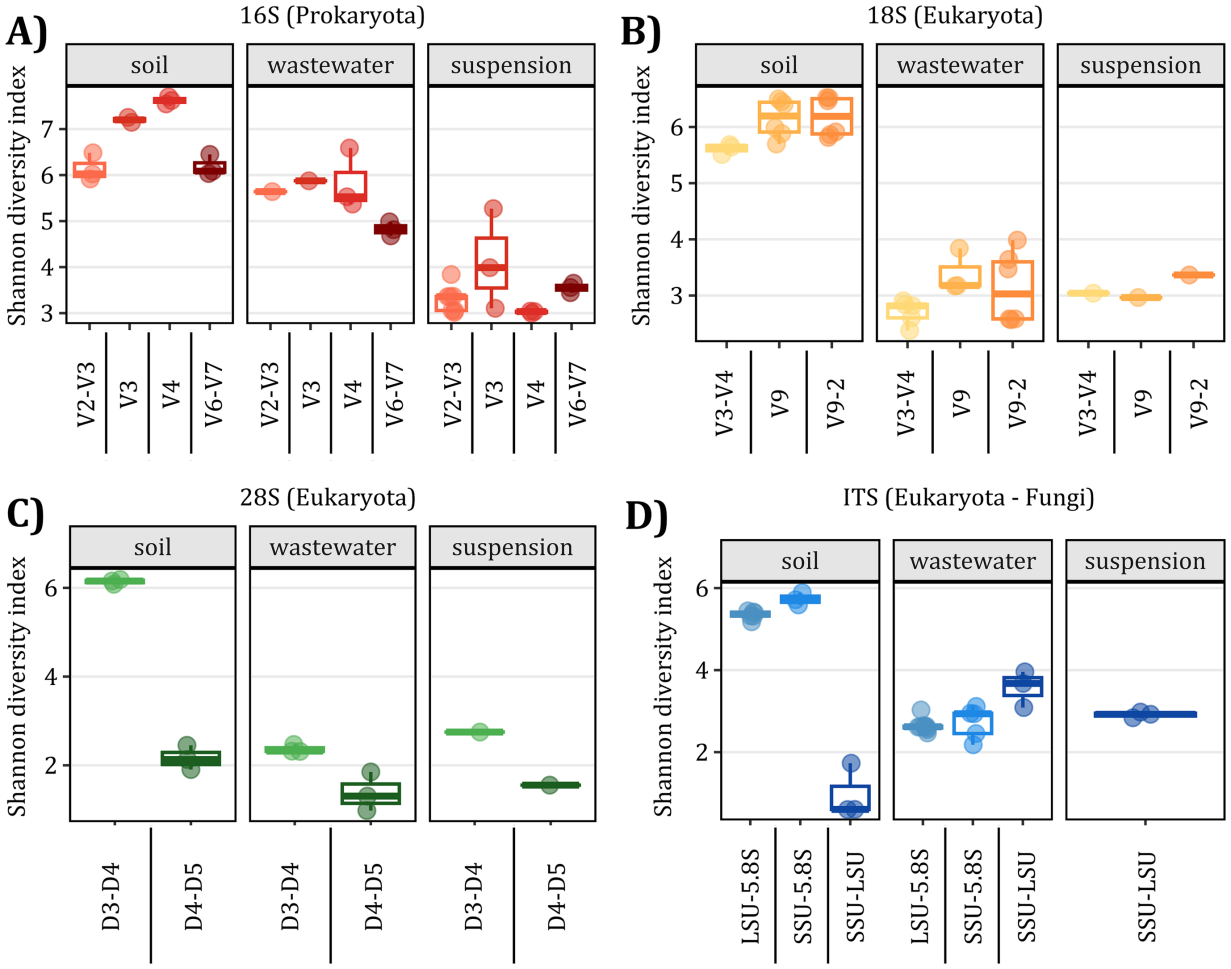
Primer-dependent differences in alpha diversity across habitats within each ribosomal marker dataset. Shannon diversity index was calculated from rarefied amplicon datasets targeting **(A)** 16S rRNA for prokaryotic communities, **(B)** 18S rRNA for eukaryotic communities, **(C)** 28S rRNA for eukaryotic communties, and **(D)** ITS regions for fungal (eukaryotic) profiling. Results are shown separately for soil (left), wastewater (middle), and suspension samples (right). Boxes represent interquartile ranges (IQR), and points indicate biological replicates.

**Figure 3:**
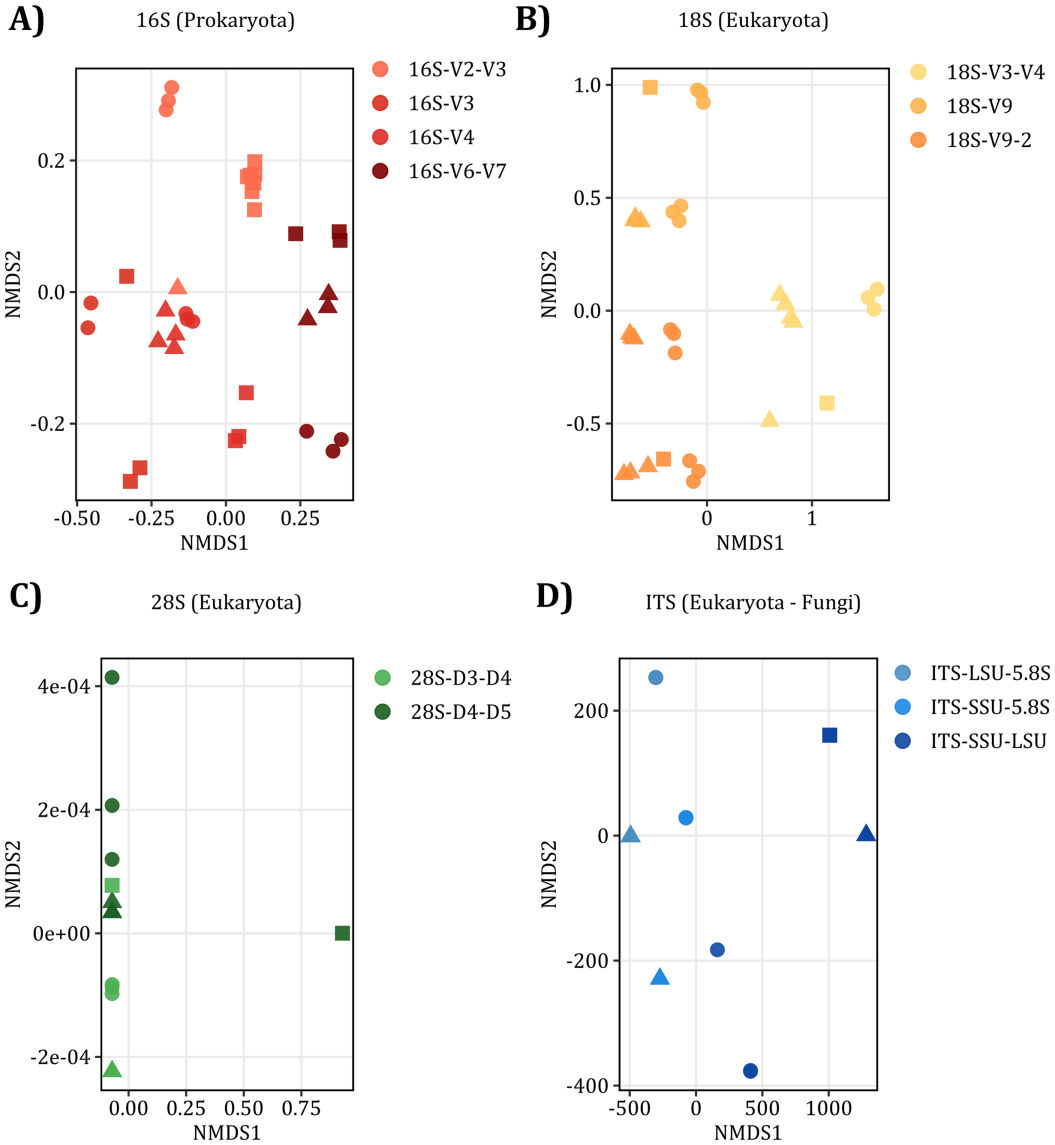
Primer-dependent differences in beta diversity within each ribosomal marker set. NMDS ordinations based on Bray–Curtis dissimilarities of relative abundance data are shown for amplicon datasets targeting **(A)** 16S, **(B)** 18S, **(C)** 28S rRNA, and **(D)** ITS regions. Samples are colored by primer set. Point shapes indicate habitat: circles, soil; triangles, wastewater; squares, suspension.

Alpha diversity was assessed using Shannon diversity after rarefaction within each marker to account for differences in sequencing depth (Figure 2; Supplementary Figure S3). Beta diversity was calculated using Bray–Curtis dissimilarities based on relative abundance data and visualized by non-metric multidimensional scaling (NMDS; Figure 3). Together, these metrics allowed us to assess whether primer choice influenced both diversity estimates and inferred community structure.

##### 3.1.1.1 Alpha diversity

Shannon diversity varied consistently among primer sets within each ribosomal marker and habitat (Figure 2). For 16S rRNA gene amplicons (Figure 2A), primers targeting the V3 and V4 regions yielded higher diversity than V2–V3 and V6–V7 primers across all habitats. These differences were most pronounced in soil samples. For 18S rRNA gene amplicons (Figure 2B), V9 primers showed higher Shannon diversity than V3–V4 primers, particularly in soil samples, whereas differences were less pronounced in suspension samples. For 28S rRNA gene amplicons (Figure 2C), primers targeting the D3–D4 region consistently resulted in higher diversity than D4–D5 primers across all habitats. ITS-based diversity estimates (Figure 2D) were strongly primer- and habitat-dependent. In soil and wastewater, primers targeting shorter ITS subregions recovered higher diversity than the SSU–LSU primer set. In suspension samples, ITS amplification was only successful with the SSU–LSU primers and resulted in comparatively low diversity.

Across all markers, suspension samples consistently showed lower diversity than soil and wastewater samples. Metagenomic profiles of these samples revealed dominance by a limited number of taxa (Supplementary Figures S4A–C), indicating reduced community complexity. This lower baseline complexity likely constrains the maximum diversity detectable by amplicon sequencing, independent of primer choice. Overall, primer choice systematically influenced diversity estimates across all markers and habitats.

##### 3.1.1.2 Beta diversity

To further compare primer performance within each marker, Bray–Curtis dissimilarities based on relative abundance data were calculated and visualized using non-metric multidimensional scaling (NMDS) ordination (Figure 3). Bray–Curtis-based NMDS ordination revealed clear clustering by primer set across all ribosomal markers (Figure 3). In many cases, samples grouped more strongly according to primer choice than habitat, indicating that primer selection substantially influences abundance-weighted community structure.

In the 16S dataset (Figure 3A), soil and wastewater samples were distributed across the ordination space primarily according to primer set rather than habitat. Primers targeting the V3 and V4 regions clustered closely together, whereas V2–V3 and V6–V7 samples formed distinct clusters. A similar separation was observed in the 18S dataset (Figure 3B), where V9-derived communities clustered separately from those obtained with V3–V4 primers. Comparable primer-dependent patterns were also evident for 28S and ITS markers (Figures 3C,D). This effect was particularly pronounced in complex habitats such as soil (circles) and wastewater (triangles), where samples amplified with different primer sets separated into distinct clusters despite originating from the same biological material.

Overall, these results show that primer choice does not alter the underlying biological community but strongly influences its representation in amplicon-based datasets, leading to consistent primer-dependent differences in observed community structure across both bacterial (16S) and eukaryotic (18S, 28S, ITS) markers.

#### 3.1.2 Shifts in observed taxonomic composition explained by primer choice

**Figure 4:**
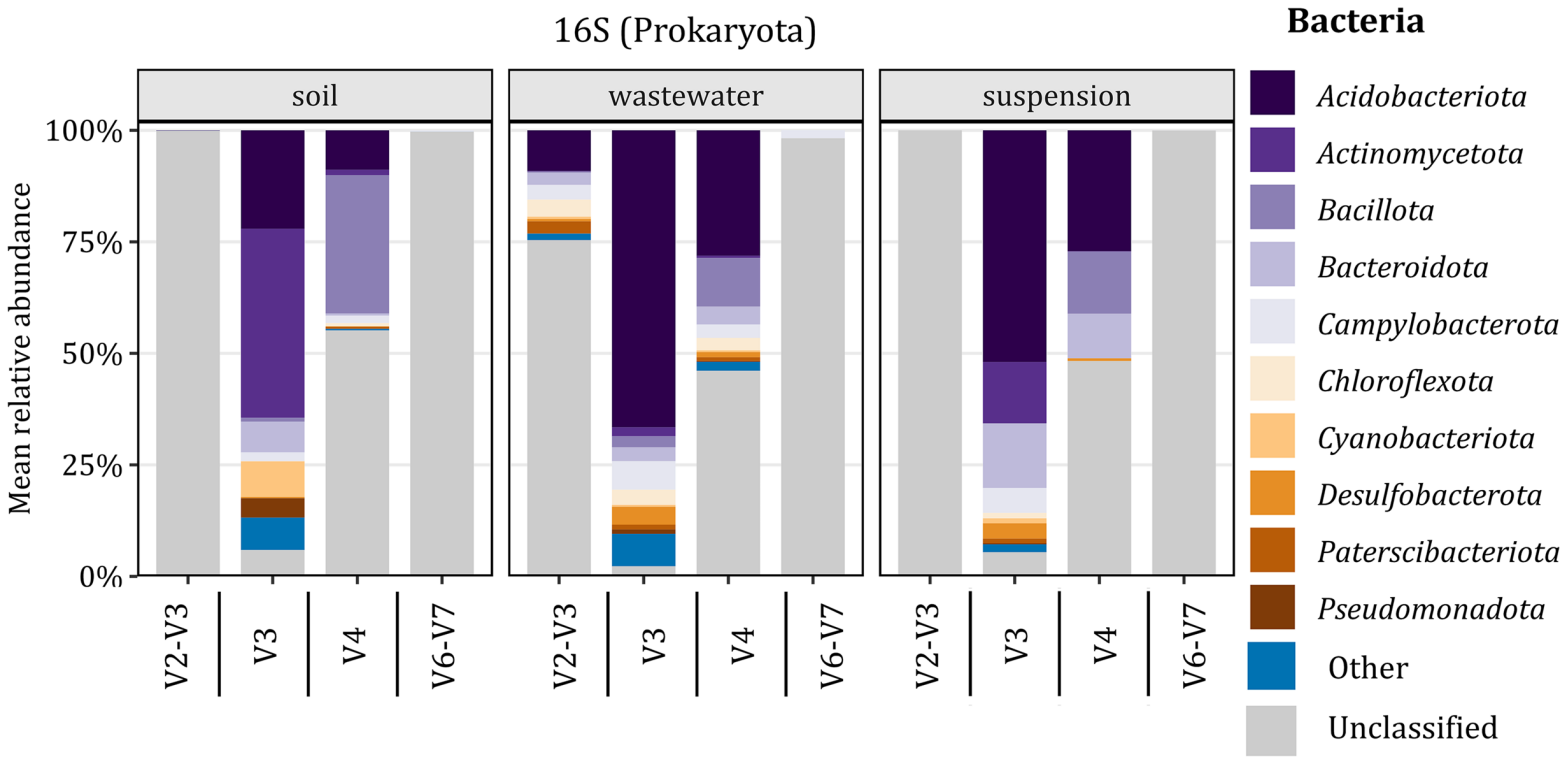
Primer-dependent differences in taxonomic composition across habitats and ribosomal markers within the prokaryotic domain. Mean relative abundances at the phylum level are shown for amplicon datasets targeting the 16S rRNA gene across soil (left), wastewater (middle), and suspension samples (right). Bars represent mean relative abundances across biological replicates for each primer set. Colors indicate major prokaryotic phyla, while grey represents unassigned sequences.

To assess how primer choice affected taxonomic profiles, we compared phylum-level community composition across habitats and marker regions (Figure 4). It is important to note that 16S rRNA gene primers target prokaryotic communities (Bacteria and Archaea), whereas 18S, ITS, and 28S primers target eukaryotic organisms. Archaeal taxa are often underrepresented due to primer mismatches and lower amplification efficiency, particularly with commonly used bacterial 16S primer sets (Moissl-Eichinger et al., 2018; Castelle et al., 2015; Mahnert et al., 2018; Eisenstein, 2018; Raymann et al., 2017). Consequently, comparisons across these markers reflect differences between domains of life rather than variation within the same biological community.

Across all ribosomal markers, taxonomic profiles differed between primer sets within the same habitat, demonstrating that primer choice affects the observed relative abundance of taxa. For 16S rRNA gene amplicons (Figure 4A), phylum-level composition depended strongly on the targeted region. In soil and wastewater samples, V3 and V4 primers recovered broader and more balanced bacterial communities, including *Acidobacteriota*, *Actinomycetota*, *Pseudomonadota*, and *Desulfobacterota*. In contrast, V2–V3 and V6–V7 primers produced less complex profiles, with reduced representation of key soil-associated phyla. These differences were accompanied by variation in the proportion of assigned reads (Supplementary Figure S5A).

**Figure 5:**
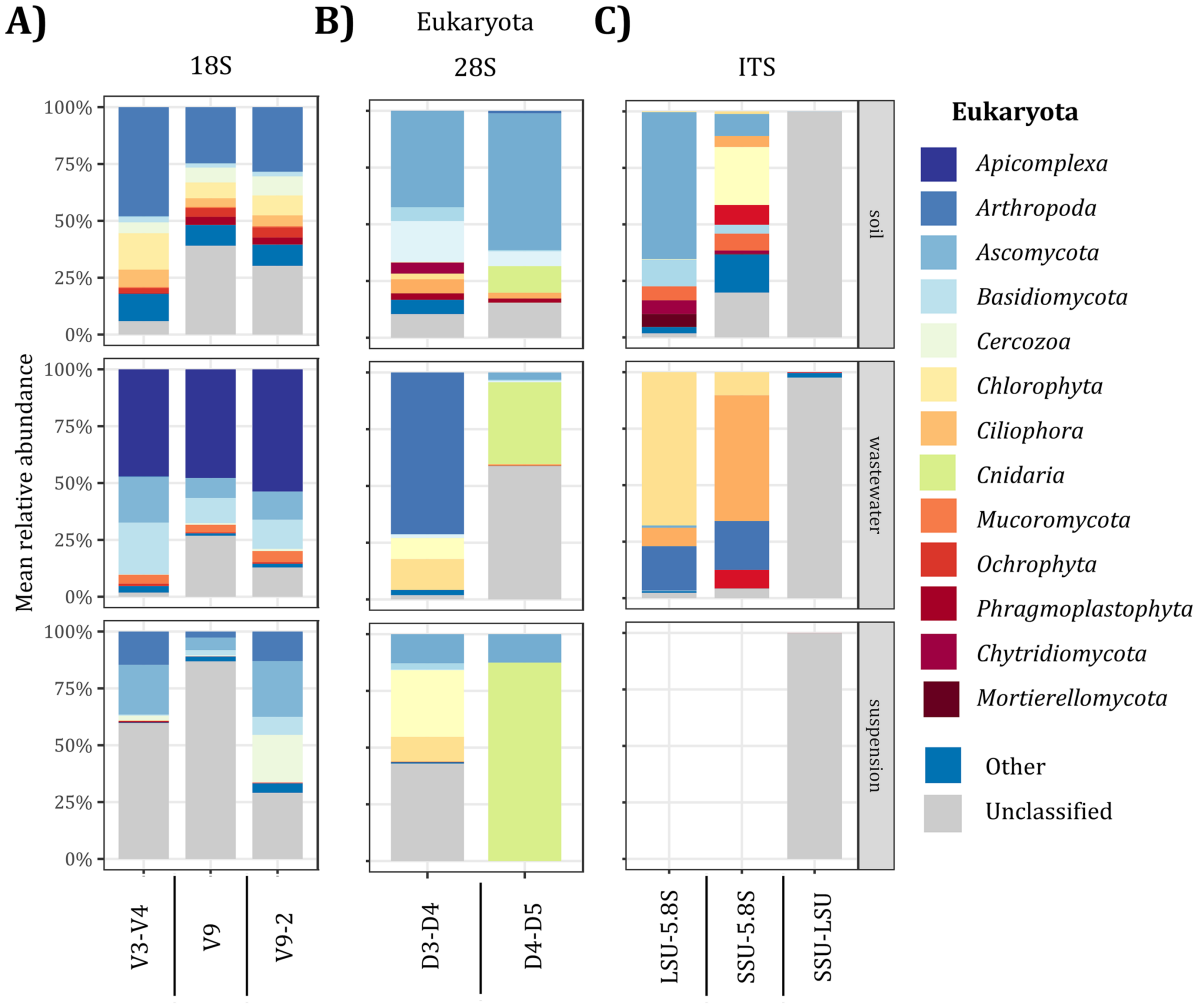
Primer-dependent differences in taxonomic composition across habitats and ribosomal markers within the Eukaryota domain. Mean relative abundances at the phylum level are shown for amplicon datasets targeting **(A)** 18S, **(B)** 28S rRNA, and **(C)** ITS regions across soil (top), wastewater (middle), and suspension samples (bottom). Bars represent mean relative abundances across biological replicates for each primer set. Colors indicate major eukaryotic phyla, while grey represents unassigned sequences.

Eukaryotic community profiles inferred from 18S rRNA gene amplicons also varied substantially among primer sets (Figure 5A). V9 primers generally recovered a broader range of taxa in soil and wastewater samples, whereas V3–V4 primers produced less diverse profiles. However, V9 primers were associated with higher proportions of unassigned reads in some habitats (Supplementary Figure S5B).

Differences between primer sets were even more pronounced for 28S rRNA gene amplicons (Figure 5B). Primers targeting the D3–D4 region yielded more diverse and informative profiles, whereas D4–D5 primers often resulted in dominance by fewer phyla and a higher fraction of unassigned sequences (Supplementary Figure S5C).

ITS amplicons showed the strongest habitat- and primer-dependent variation (Figure 5C). In soil and wastewater samples, primers targeting shorter ITS subregions recovered diverse fungal communities with comparatively high assignment rates. In contrast, the SSU–LSU primer set produced a large proportion of unassigned reads and less resolved profiles (Supplementary Figure S5D). In suspension samples, ITS amplification was largely limited to SSU–LSU primers and resulted predominantly in unassigned sequences, consistent with the low eukaryotic representation observed in the corresponding metagenomes (Supplementary Figure S4A).

Across all markers, differences in taxonomic composition were closely linked to the proportion of assigned versus unassigned reads (Supplementary Figure S5). Primer sets with higher assignment rates generally showed better agreement with metagenomic profiles (Supplementary Figures S4B,C), whereas those with a high fraction of unassigned reads provided lower ecological resolution. Comparisons of the most and least abundant families across primer sets (Supplementary Figures S6, S7) further showed that both dominant and low-abundance taxa were inconsistently recovered, indicating that primer choice directly affects taxon detectability.

Taken together, these results demonstrate that primer choice influences more than sequencing depth or diversity estimates. It substantially affects the perceived community structure by altering taxon representation through preferential amplification and differences in taxonomic assignment. Consequently, interpretations of microbial dominance and habitat composition can vary depending on the selected primer set, even when analyzing identical biological samples. Importantly, these differences reflect methodological biases in detection and representation rather than true biological shifts in community composition.

#### 3.1.3 Concordance between amplicon and metagenomic profiles

**Figure 6:**
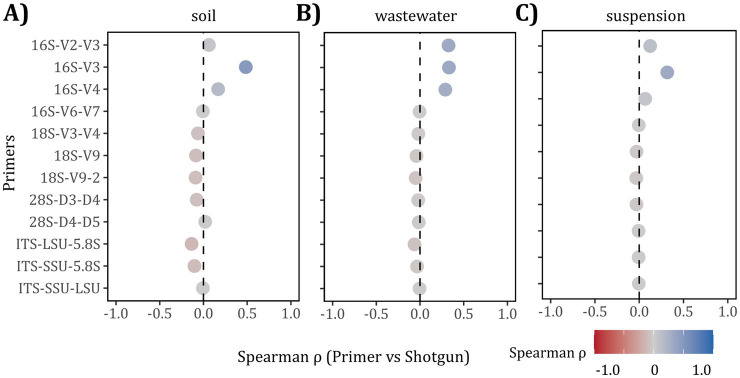
Concordance between amplicon-based and metagenomic taxonomic profiles. Spearman rank correlations (*ρ*) between relative taxonomic abundances derived from amplicon sequencing and metagenome-derived ribosomal marker profiles are shown for **(A)** soil, **(B)** wastewater, and **(C)** suspension samples. Each point represents the correlation for one primer set, calculated at the family level. Colors indicate the direction and strength of the correlation, with blue representing positive and red negative correlations. The dashed vertical line indicates zero correlation.

To evaluate how accurately primer-specific amplicon profiles reflected compared with PCR-independent ribosomal marker profiles, we calculated Spearman rank correlations (Schober et al., 2018) between family-level relative abundances derived from amplicon sequencing and corresponding metagenome-derived ribosomal marker profiles (Figure 6). This analysis was intented to assess primer-dependent agreement with a marker-matched metagenomic reference rather than to infer complete whole-community composition for genome-wide shotgun data.

Across all habitats, concordance varied substantially among markers and primer sets. For bacterial profiling, 16S rRNA gene amplicons showed the highest agreement with metagenomic profiles, particularly for primers targeting the V3 and V4 regions in soil samples (Figure 6A, top). These primers exhibited moderate to strong correlations, indicating improved recovery of dominant bacterial taxa.

In contrast, eukaryotic markers (18S, 28S, ITS) showed generally weaker correlations across habitats. Although 18S V9 primers captured a broader eukaryotic signal, agreement with metagenomic profiles remained limited, especially in wastewater and suspension samples (Figures 6B,C). Correlations were lowest in suspension samples across all markers (Figure 6C). Even 16S primers showed only moderate agreement, consistent with the dominance of a few taxa in these communities. In such cases, small shifts in relative abundance can strongly influence correlation values.

Importantly, neither higher sequencing depth (Supplementary Figure S3) nor higher alpha diversity (Figure 2) consistently translated into improved agreement with metagenomic profiles (Supplementary Figure S4). For example, some primer sets, such as 16S-V4 or 18S-V9, yielded diverse community profiles (Figures 2A,B) but showed comparatively weak concordance (Figure 6), indicating that diversity alone does not guarantee accurate community representation.

Overall, primer choice influenced diversity estimates, taxonomic composition, and agreement with metagenomic data. Although 16S-V3 showed the most consistent performance under the tested conditions, no primer set fully reconstructed community structure. Shotgun metagenomics therefore provided the most comprehensive reference for community profiling in this study.

However, despite its advantages, shotgun metagenomic sequencing remains comparatively resource-intensive in terms of cost, data processing, and computational requirements, which can limit its applicability for large-scale or high-throughput studies (Ranjan et al., 2016; Segura et al., 2025). In contrast, amplicon sequencing offers a cost-effective, scalable, and methodologically accessible approach for microbial community analysis. Based on the benchmarking results, and considering these practical advantages, the best-performing primer set (16S-V3) was selected for downstream analysis of MESIF-based enrichment experiments.

### 3.2 Application of optimized primer to soil-derived MESIF chip sampling

#### 3.2.1 Cultivation-induced changes in diversity

Given the pronounced primer-dependent effects observed during benchmarking, we next applied the best-performing primer set (16S-V3) to analyze bacterial communities in a soil-derived MESIF enrichment system. While shotgun metagenomics provided a comprehensive reference for community profiling, its higher cost and computational demands can limit its applicability for large-scale, longitudinal, or high-replicate experimental designs. Amplicon sequencing, in contrast, enables scalable analysis of such systems and was therefore used to monitor enrichment dynamics over time.

**Figure 7:**
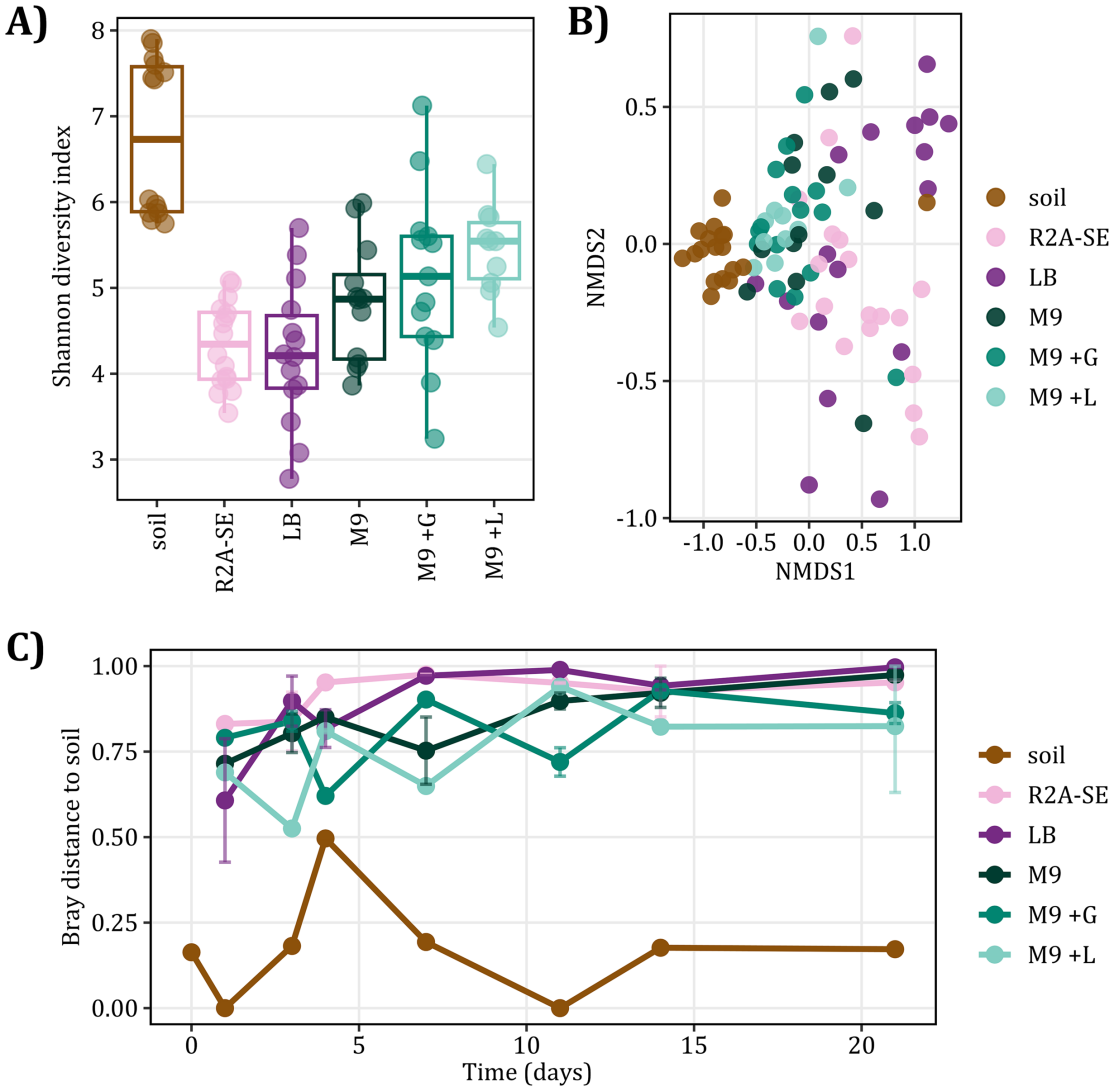
Medium-driven shifts during soil-derived MESIF enrichment. **(A)** Shannon diversity across soil control and enrichment media (LB, M9, M9 + G, M9 + L, and R2A-SE) analyzed using the 16S-V3 primer set. Boxes represent interquartile ranges (IQR), with points indicating individual biological replicate samples collected across the time series. For MESIF treatments, *n* = 3 independent chips were analyzed per medium and time point. **(B)** NMDS ordination based on Bray–Curtis dissimilarities showing community structure across soil and enrichment media. **(C)** Bray–Curtis distance to the native soil community at day 1 across the incubation period (days 1–21).

MESIF chips were supplemented with five different cultivation media to assess selective enrichment relative to native soil communities. Communities were monitored over a 21-day incubation period, with native soil samples processed in parallel as habitat controls. To evaluate how cultivation within MESIF chips altered soil-derived communities, we assessed alpha and beta diversity and quantified divergence from the native soil baseline, using day 1 as the reference point (Figure 7).

Shannon diversity (Figure 7A) differed markedly between native soil and MESIF-derived communities. Soil controls consistently exhibited the highest alpha diversity, reflecting the complex and heterogeneous structure of the native community. In contrast, all MESIF treatments showed reduced diversity. Among these, M9 supplemented with lactose (M9 + L) generally maintained the highest diversity, followed by M9 + G and M9 without an additional carbon source. R2A-SE and LB showed the lowest diversity. These differences were not driven by sequencing depth (Supplementary Figure S8A), and observed ASV richness clearly distinguished native soil from MESIF samples (Supplementary Figure S8B).

Bray–Curtis-based NMDS ordination revealed clear separation between native soil samples and MESIF communities (Figure 7B). Soil samples clustered tightly, indicating a relatively stable community structure over time. In contrast, MESIF samples formed a broader and partially overlapping cluster, reflecting a pronounced shift in community composition relative to the native soil. Although samples from the same cultivation medium tended to cluster closer together, separation between media remained limited. This pattern was consistent across additional diversity metrics (Supplementary Figure S8), indicating that enrichment effects were stronger than medium-specific differences at the community level.

To quantify the magnitude of community change, Bray–Curtis distances to the native soil baseline (day 1) were calculated for each treatment over time (Figure 7C). MESIF communities diverged rapidly from the native soil within the first days of incubation and remained distinct throughout the experiment. In contrast, soil controls maintained low distances, confirming temporal stability. Minor temporal fluctuations were observed within MESIF treatments, but no convergence toward the native soil community was detected.

Together, these results demonstrate that cultivation within MESIF chips leads to rapid and sustained divergence from native soil communities. During the 21-day incubation period, MESIF cultivation produced a stronger taxonomic divergence from native soil than the medium-specific differences detected among MESIF treatments. This pattern is consistent with early colonization of the structured MESIF matrix by fast-growing, surface-associated taxa, a process expected to favor organisms with strong surface-attachment and biofilm-forming capacities. However, the short incubation time and 16S rRNA gene-based resolution do not allow us to exclude later-emerging substrate-specific selection or functional differences among similar taxonomic groups.

#### 3.2.2 Medium-associated taxonomic composition

**Figure 8:**
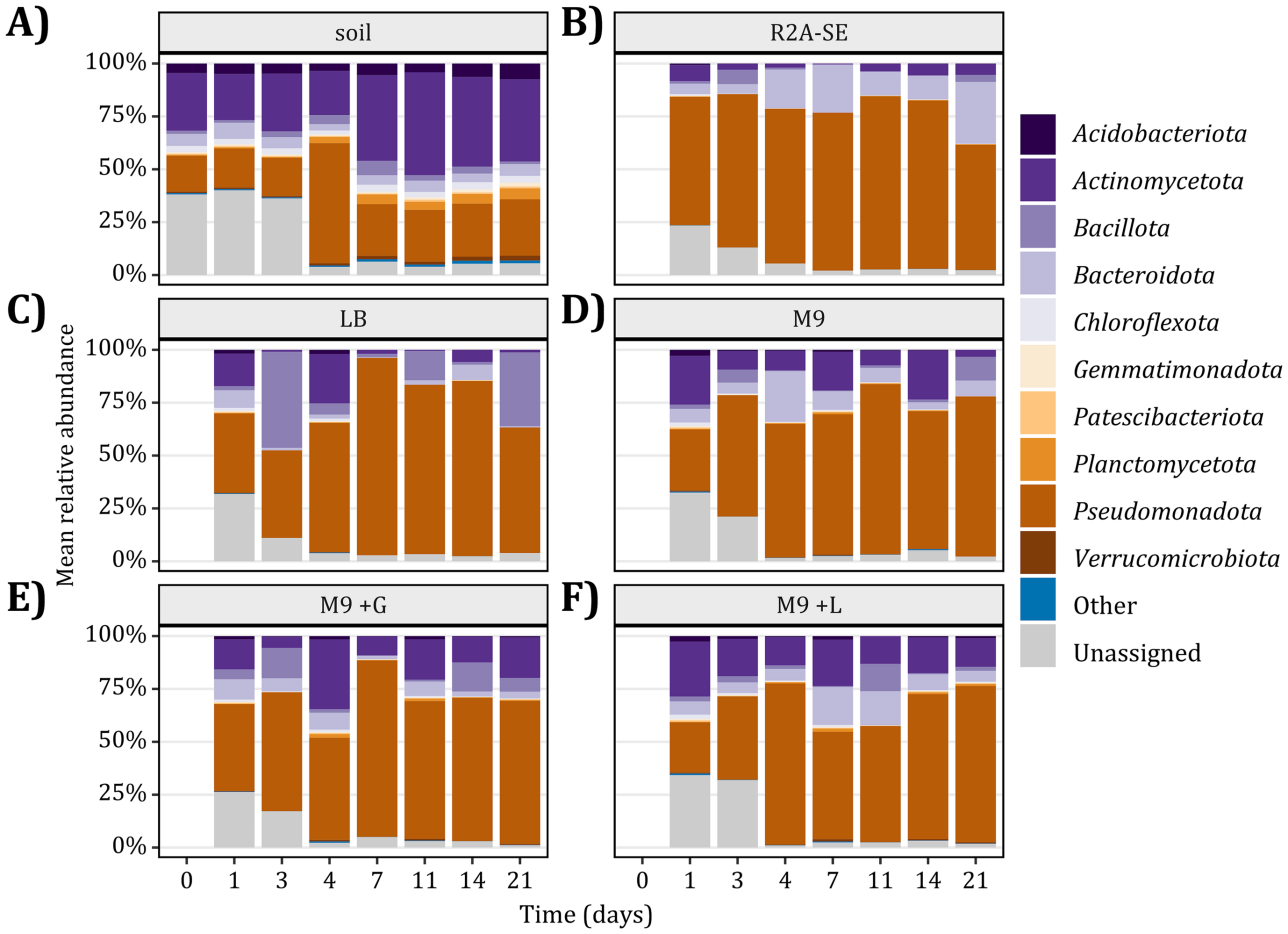
Temporal shifts in phylum-level community composition. Mean relative abundances of dominant phyla across incubation time points for **(A)** soil, **(B)** LB, **(C)** M9, **(D)** M9 + G, **(E)** M9 + L, and **(F)** R2A-SE treatments. Bars represent mean relative abundances across biological replicates.

To investigate how cultivation media influenced community composition over time, we examined bacterial phylum-level relative abundances across all treatments (Figure 8).

Native soil communities displayed a diverse and relatively stable composition, dominated by *Acidobacteriota*, *Actinomycetota*, and *Pseudomonadota*, with only minor temporal variation (Figure 8A).

In contrast, all MESIF treatments showed pronounced shifts in taxonomic composition compared to soil. Across all media, reads assigned to the phylum *Pseudomonadota* increased rapidly during incubation and became the dominant phylum at later time points. Concurrently, typical soil-associated taxa such as *Acidobacteriota* declined under all conditions.

Although overall community trajectories were similar across media, some differences were observed. LB and R2A-SE showed a rapid and strong dominance of *Pseudomonadota* (Figures 8B,C), whereas M9-based media (with or without glucose or lactose) retained slightly higher proportions of additional phyla over time (Figures 8D–F).

Across all treatments, communities became less diverse and increasingly dominated by a limited number of taxa. The proportion of unassigned reads varied across time points and treatments (Supplementary Figure S9). Because unassigned reads may result from incomplete reference database coverage, ambiguous taxonomic placement, low-quality sequences, or residual artefacts, these patterns were interpreted cautiously and were not used as direct evidence of community shifts.

Together, these results demonstrate that MESIF cultivation consistently alters community composition relative to native soil during the early enrichment phase. While the strength of enrichment varied among media, the overall phylum-level patterns were broadly similar, indicating that the cultivation system exerts a stronger influence on community structure than the specific medium composition. At the same time, the observed temporal dynamics, including the progressive dominance of specific taxa and the corresponding decline in unassigned sequences, suggest that MESIF chips not only enrich selected microbial groups but also capture reproducible shifts in community structure over time. This indicates that, although MESIF chips selectively enrich specific taxa, they can still be used to monitor and track broader community-level changes under controlled conditions.

#### 3.2.3 Differentially enriched taxa reveal selective pressures in structured matrices

**Figure 9:**
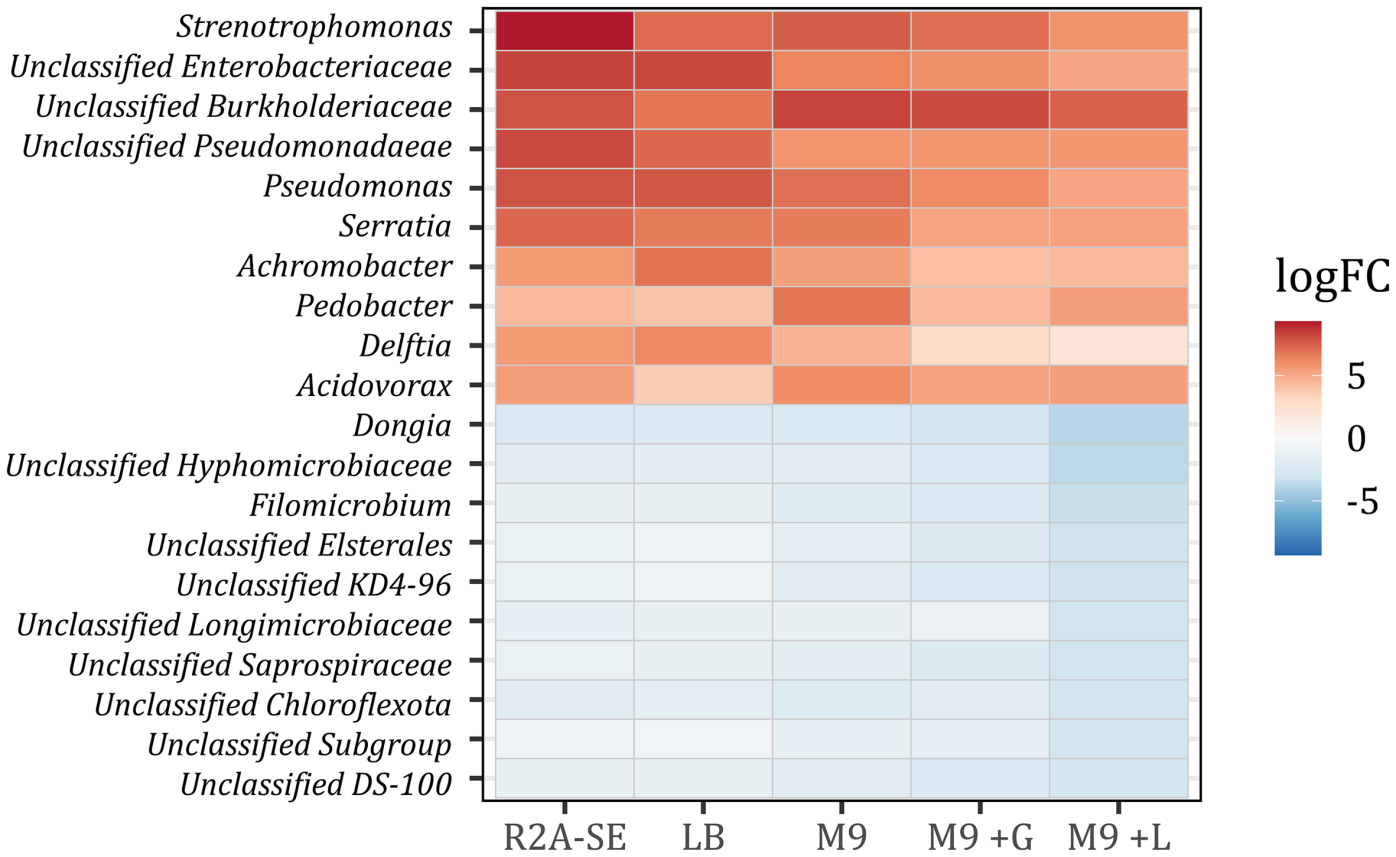
Differentially enriched genera across cultivation media relative to soil. Heatmap showing log_2_ fold changes (logFC) of the top 10 significantly enriched or depleted genera in LB, M9, M9 + G, M9 + L, and R2A-SE treatments. Positive values (red) indicate enrichment relative to soil, whereas negative values (blue) indicate depletion. The color scale is centered at zero and ranges from −5 to +5 log_2_FC, with values outside this range clipped to the scale limits. Only genera with statistically significant differential abundance after Benjamini–Hochberg FDR correction (*p* < 0.05) are shown.

To identify taxa significantly enriched or depleted in MESIF chips relative to native soil, we performed differential abundance analysis at the genus level using ANCOM-BC. The 10 most strongly enriched and depleted genera were visualized based on their log2 fold changes (Figure 9).

Across all enrichment treatments, several genera belonging to the phylum Pseudomonadota were consistently enriched relative to soil. These included *Pseudomonas*, *Stenotrophomonas*, unclassified members of *Pseudomonadaceae*, and *Burkholderiaceae*, as well as *Achromobacter*, *Delftia*, and *Acidovorax*. In contrast, several soil-associated and poorly characterized taxa, including members of *Chloroflexota* and other unclassified groups, were consistently depleted.

Enrichment patterns were largely shared across all media, including LB, M9, and carbon-supplemented M9 variants. No clear medium-specific signature linked to a particular carbon source was observed at the genus level. Although the magnitude of enrichment differed among treatments, the identity of enriched taxa remained broadly consistent across media. This suggests that early community restructuring was strongly influenced by colonization of the structured MESIF matrix.

This pattern is consistent with the structured environment provided by MESIF chips, which promotes surface colonization and biofilm formation (Zoheir et al., 2022; Sauer et al., 2022). Several enriched genera, including *Pseudomonas* and *Stenotrophomonas*, are known for rapid growth and strong biofilm-forming capacity (Klausen et al., 2003; Bonaventura et al., 2004; Isom et al., 2022). Given the relatively short incubation period, these results likely reflect early stages of community assembly, during which fast-growing taxa expand before longer-term stabilization occurs (Nemergut et al., 2013).

Together, these findings indicate that MESIF cultivation preferentially selects for bacteria adapted to nutrient-rich, surface-associated conditions, while reducing the representation of typical soil-associated taxa. Although selection was not strongly carbon-specific, the system enabled consistent and reproducible enrichment under controlled conditions, highlighting the dominant role of physical structure and surface-associated growth in shaping enrichment outcomes.

## 4 Conclusion and outlook

In this study, we demonstrated that primer choice introduces consistent and substantial differences in amplicon-based microbial community profiles across habitats and ribosomal marker genes. These differences affected relative abundance patterns, diversity estimates, and agreement with amplification-independent metagenomic data, highlighting that primer selection is a critical determinant of ecological interpretation.

The metagenome-derived ribosomal marker profiles provided a PCR-independent, marker-matched reference for evaluating primer-dependent representation of ribosomal community profiles. However, this reference does not capture the full taxonomic information available from whole-metagenome shotgun data and remains subject to biases associated with assembly, marker recovery, ribosomal gene copy number, and database-dependent taxonomic assignment. However, its higher cost and computational requirements can limit its applicability for large-scale, longitudinal, or high-replicate experimental designs. In contrast, amplicon sequencing represents a cost-effective, scalable, and methodologically accessible approach for high-throughput community analysis, provided that primer-specific biases are carefully considered and, where possible, benchmarked against amplification-independent data.

Using this benchmarking framework, we identified a best-performing bacterial primer set under the tested conditions and applied it to a structured soil enrichment system. Within MESIF chips, bacterial communities rapidly diverged from native soil and converged toward less diverse assemblages dominated by a limited number of taxa. Although the strength of enrichment varied among media, community trajectories were largely similar at the 16S rRNA gene level during the 21-day incubation period. This indicates that early taxonomic restructuring was strongly associated with MESIF-based structured cultivation, while medium-specific effects were comparatively less resolved under these conditions. Importantly, this conclusion is limited to short-term taxonomic enrichment patterns. The present data do not exclude substrate-specific functional selection, strain-level differentiation, or later successional divergence among media. Longer incubation periods combined with metagenomic, metatranscriptomic, or isolate-based analyses will be required to determine whether different substrates select for distinct functional traits within apparently similar taxonomic groups.

While carbon supplementation did not result in clearly distinct taxonomic trajectories, defined media such as M9 without an additional carbon source may provide a controlled baseline for detecting more subtle ecological shifts with reduced nutrient-driven bias. The consistent and reproducible enrichment patterns observed here further suggest that MESIF systems can serve not only as cultivation platforms but also as controlled model systems for tracking microbial community dynamics over time. However, the enrichment of environmentally widespread biofilm-forming taxa, including groups that may contain opportunistic pathogenic species or strains, should be acknowledged when interpreting MESIF-based enrichment outcomes. Although no pathogenicity or virulence potential was assessed in this study, noting the presence of such taxa is important for future application-oriented studies and for contextualizing appropriate handling considerations under controlled laboratory conditions.

Beyond their experimental relevance, these findings have direct implications for applied microbial ecology. Improved primer selection and systematic benchmarking can enhance the comparability and reliability of microbial community analyses across studies, which is particularly important for applications such as environmental monitoring, wastewater treatment processes, and biotechnological production systems. In addition, controlled environment agriculture (CEA) systems increasingly rely on integrated, multi-modal monitoring approaches that combine physicochemical sensors with advanced sensing technologies to capture biological processes across trophic levels (Werner et al., 2026). Within such systems, microbial community profiling represents a critical but often underresolved component (Glockow et al., 2024), and may benefit from robust and scalable sequencing-based approaches to complement existing monitoring frameworks.

At the same time, structured cultivation systems such as MESIF chips provide a framework for reproducible enrichment and targeted recovery of functionally relevant microorganisms, including previously uncultivated taxa and members of the so-called microbial dark matter (MDM) (Lock, 2015). By combining controlled cultivation with optimized molecular profiling, such systems may help bridge the gap between community-level observations and functional microbial applications.

Future work should extend incubation periods to assess longer-term successional dynamics and evaluate whether stronger substrate-specific selection emerges under prolonged cultivation. Although PCR negative controls were included, sequenced extraction blanks and unincubated MESIF-only controls were not included in this experiment. Future MESIF-based amplicon studies should incorporate these controls to more explicitly assess potential reagent-, extraction-, or material-associated background contamination, particularly during early colonization stages or in low-biomass settings. Furthermore, future benchmarking studies should compare primer performance against complementary WMS profiling approaches, including clade-specific marker-gene profilers and genome-resolved metagenomics. More broadly, integrating primer benchmarking with application-driven experimental designs will be essential to link methodological choices more directly to functional and ecological outcomes.

## Data Availability

The datasets presented in this study can be found in online repositories. The names of the repository/repositories and accession number(s) can be found at: https://www.ncbi.nlm.nih.gov/, PRJNA1442665.
