# Supplementary material for: Primer choice shapes microbial community interpretation across habitats and informs short-term structured enrichment in environmental and applied systems
Source: Front Microbiol. 2026 May 29;17:1838890. doi: 10.3389/fmicb.2026.1838890 (PMC13260388; doi:10.3389/fmicb.2026.1838890)
Supplement: Supplementary file 1 [file Data_Sheet_1.docx]

Supplementary Material

## Primer choice shapes microbial community interpretation across habitats and informs structured enrichment in environmental and applied systems

**Supplementary Figures**


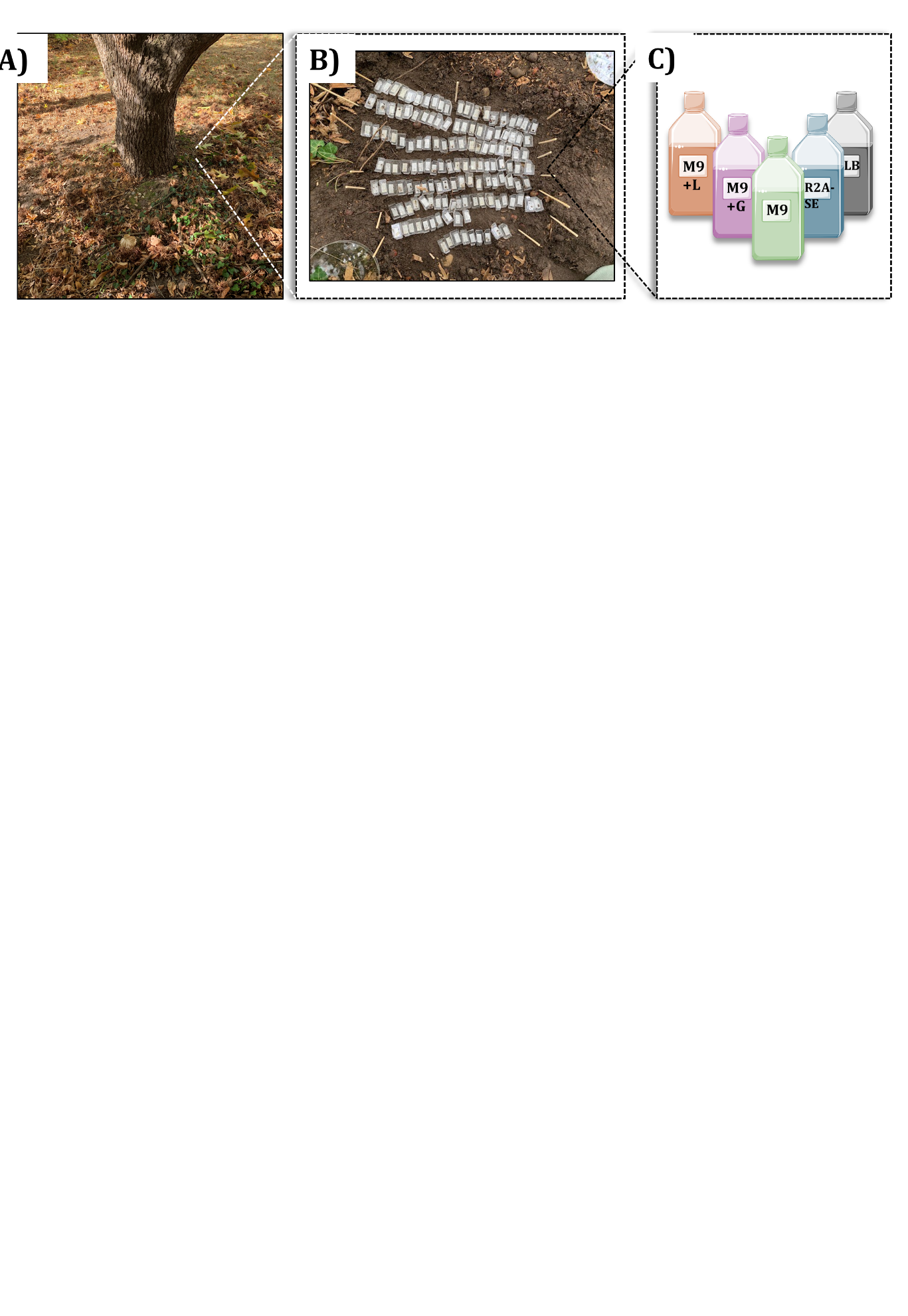


Figure S 1: Experimental setup for soil-based MESIF enrichment. (A–B) Photographs of the soil sampling site at KIT Campus North (A) and placement of MESIF chips within the soil matrix (B). (C) Schematic overview of MESIF chips supplemented with five cultivation media: M9 without an additional carbon source (M9), M9 supplemented with glucose (M9 + G), M9 supplemented with lactose (M9 + L), R2A-SE, and LB medium


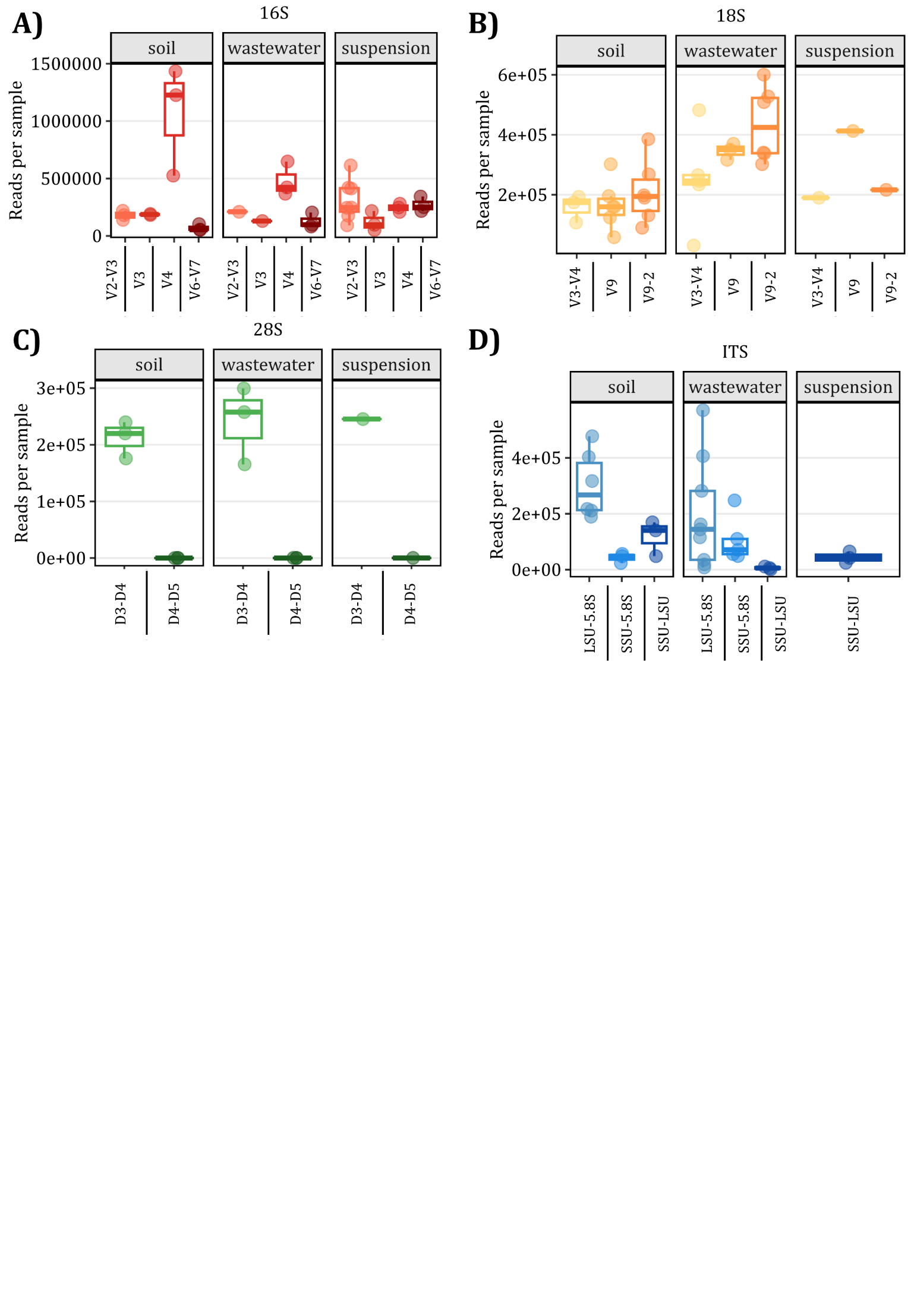


Figure S2: Sequencing depth across primer sets. Distribution of total read counts per sample for each primer set after quality filtering and processing. Panels show amplicon datasets targeting (A) 16S, (B) 18S, (C) 28S rRNA, and (D) ITS regions.


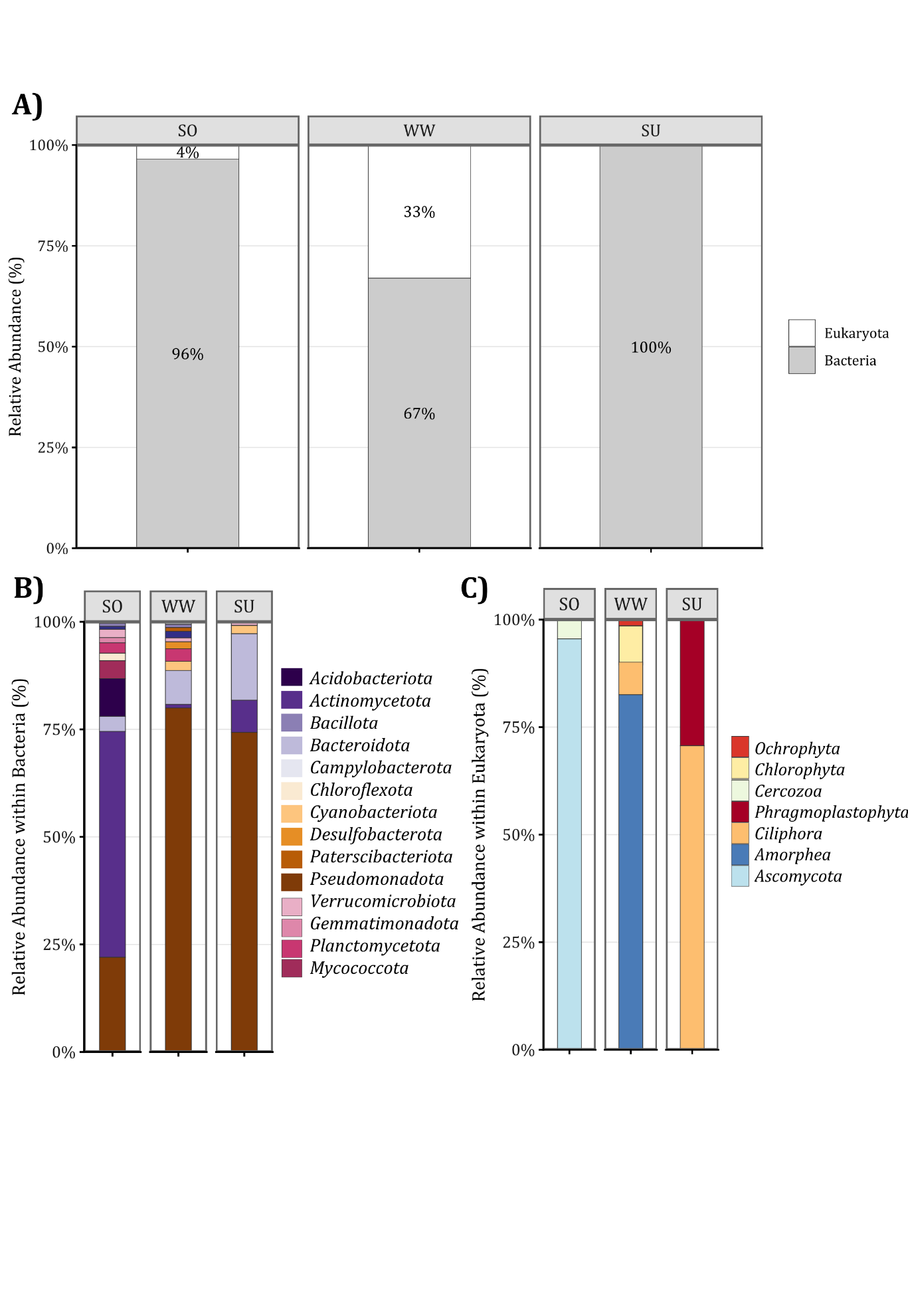


Figure S3: Phylum-level taxonomic composition of metagenomic samples. Relative abundances of microbial taxa derived from shotgun metagenomic sequencing across three habitats: soil (SO), wastewater (WW), and suspension (SU). Taxonomic profiles were generated by ribosomal marker extraction using MDMCleaner followed by classification with SINA. (A) Overall community composition showing the relative contributions of Bacteria and Eukaryota. (B) Phylum-level composition within the bacterial fraction, normalized to 100% per habitat. (C) Phylum-level composition within the eukaryotic fraction, normalized to 100% per habitat.


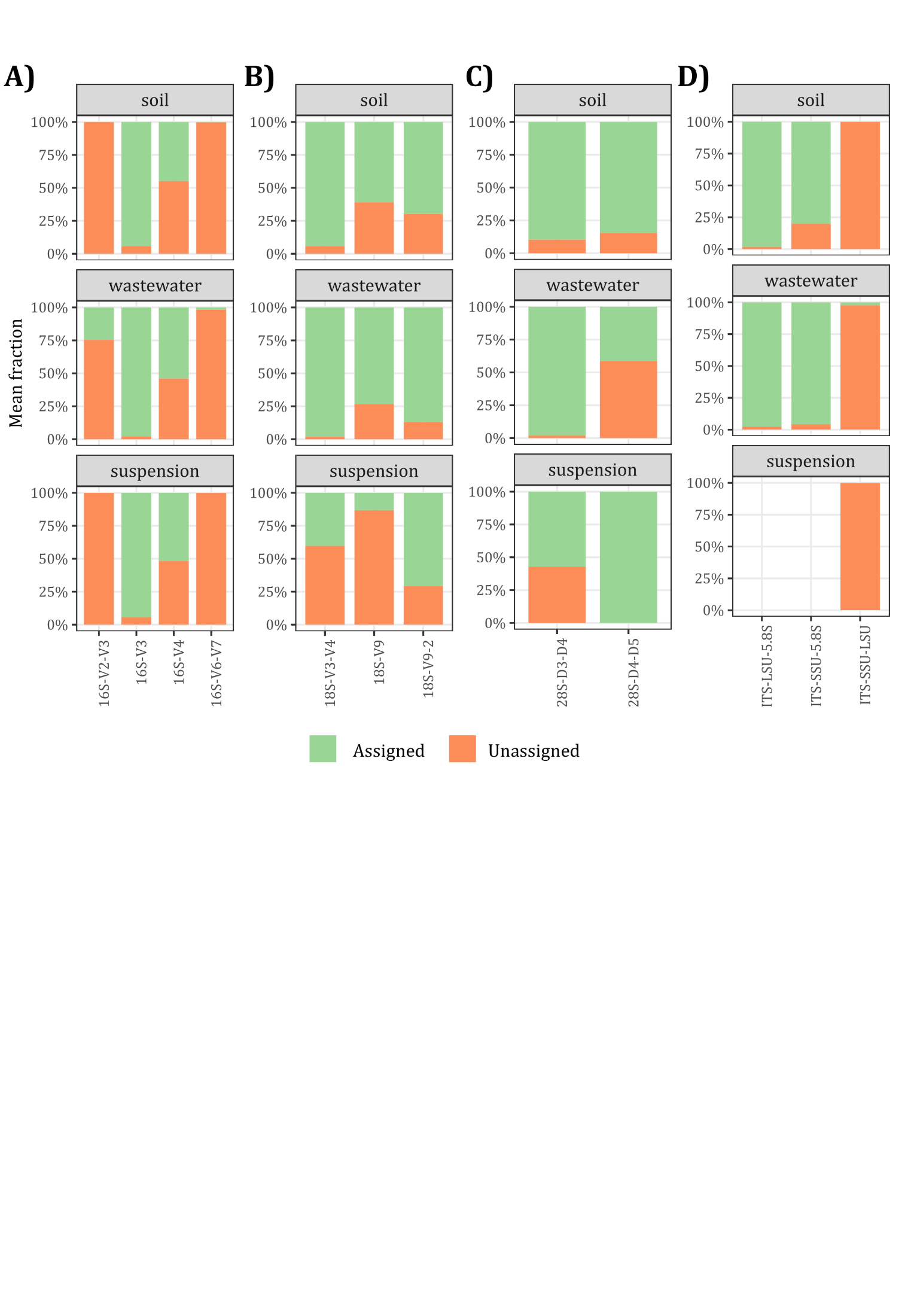


Figure S4: Proportion of taxonomically assigned and unassigned reads across primer sets. Relative proportions of reads assigned to a taxonomic rank versus unassigned reads are shown for amplicon datasets targeting (A) 16S, (B) 18S, (C) 28S rRNA, and (D) ITS regions.


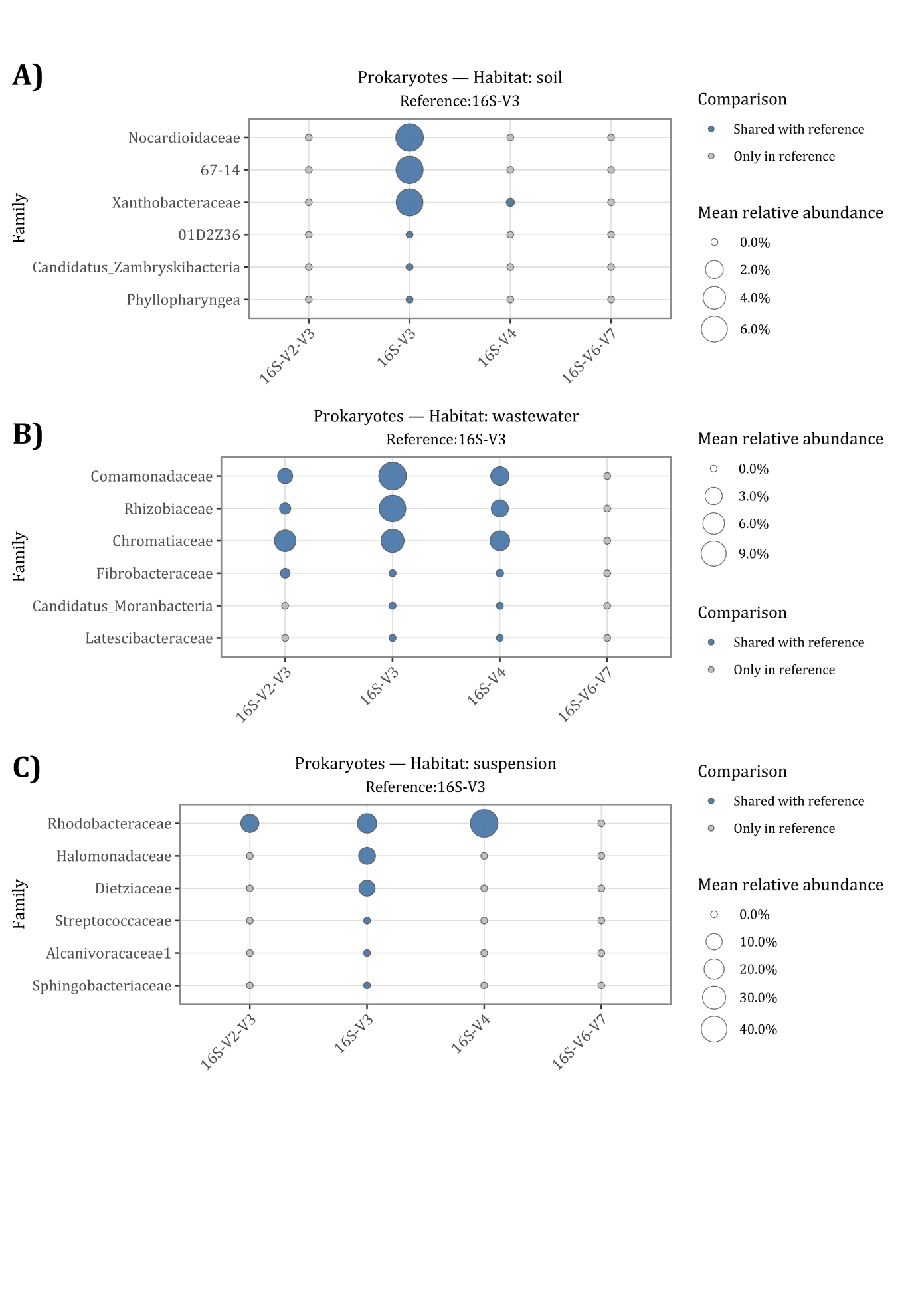


Figure S5: Primer-dependent detection of prokaryotic taxa across habitats. Dot plots showing taxon detection and relative abundance for (A) soil, (B) wastewater, and (C) suspension samples. Taxa were selected based on the reference primer 16S-V3, including the three most abundant and three least abundant (but present) taxa per habitat. Each column represents a primer set, and each row a taxon. Dot size indicates mean relative abundance (%) across samples within each habitat and primer set. Dot color indicates whether a taxon is shared with the reference primer (blue) or detected only by the reference primer (grey).


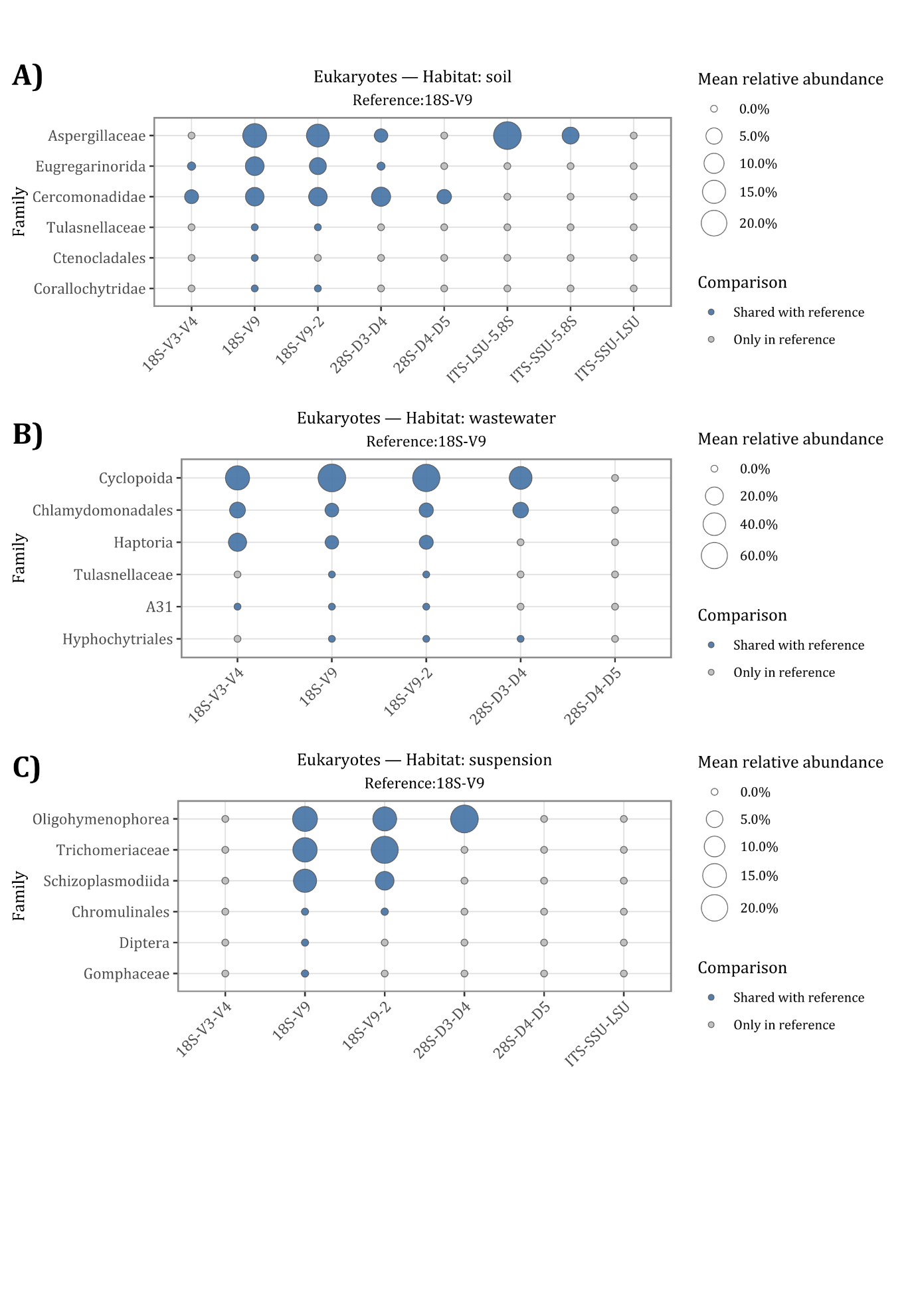


Figure S6: Primer-dependent detection of eukaryotic taxa across habitats. Dot plots showing taxon detection and relative abundance for (A) soil, (B) wastewater, and (C) suspension samples. Taxa were selected based on the reference primer 18S-V9, including the three most abundant and three least abundant (but present) taxa per habitat. Each column represents a primer set, and each row a taxon. Dot size indicates mean relative abundance (%) across samples within each habitat and primer set. Dot color indicates whether a taxon is shared with the reference primer (blue) or detected only by the reference primer (grey).


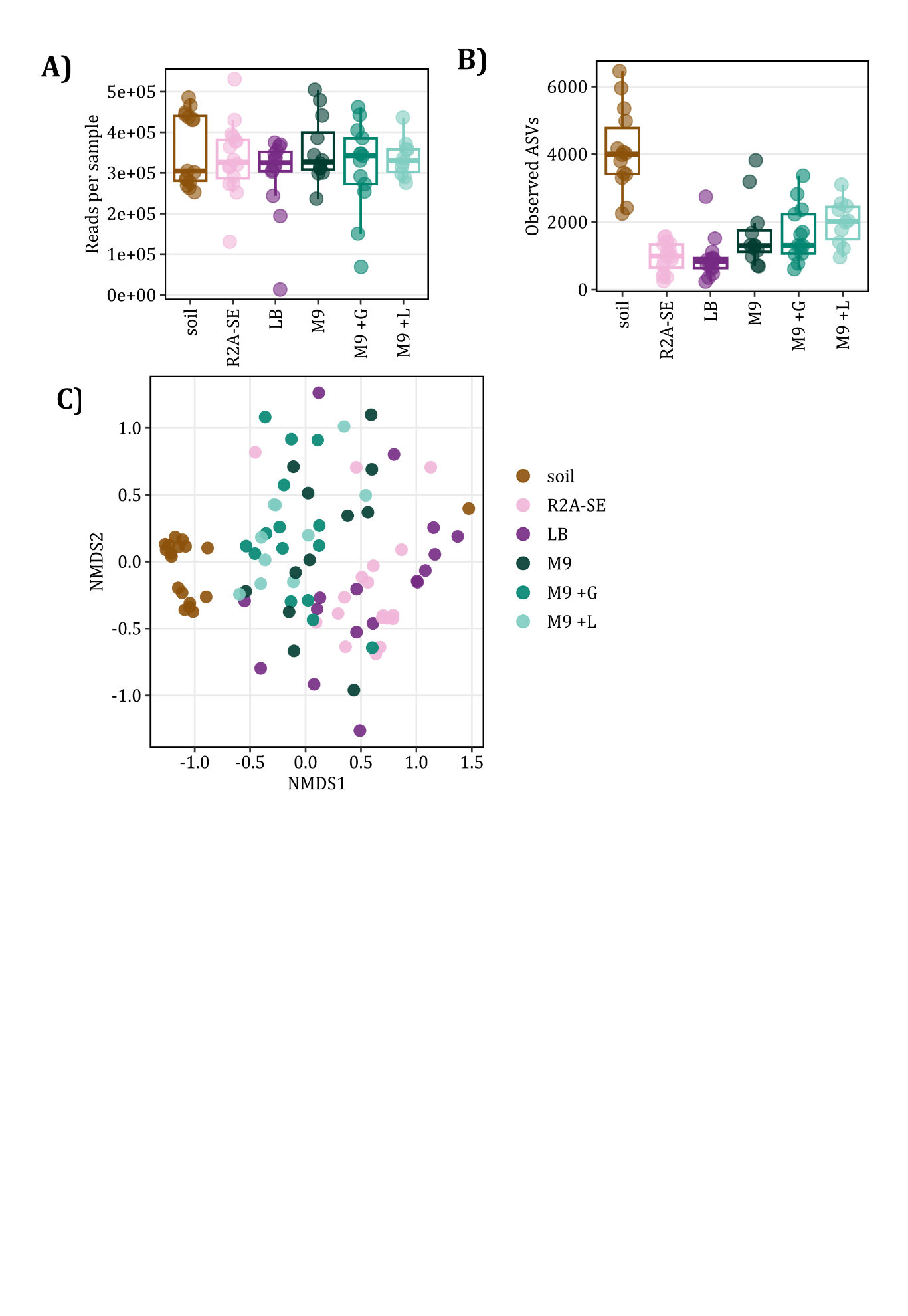


Figure S7: Diversity metrics and community structure of soil and MESIF samples. (A) Sequencing depth distribution (reads per sample) across soil and cultivation treatments. (B) Observed ASVs per sample as a measure of alpha diversity. (C) NMDS ordination based on Jaccard distances showing community structure across treatments.


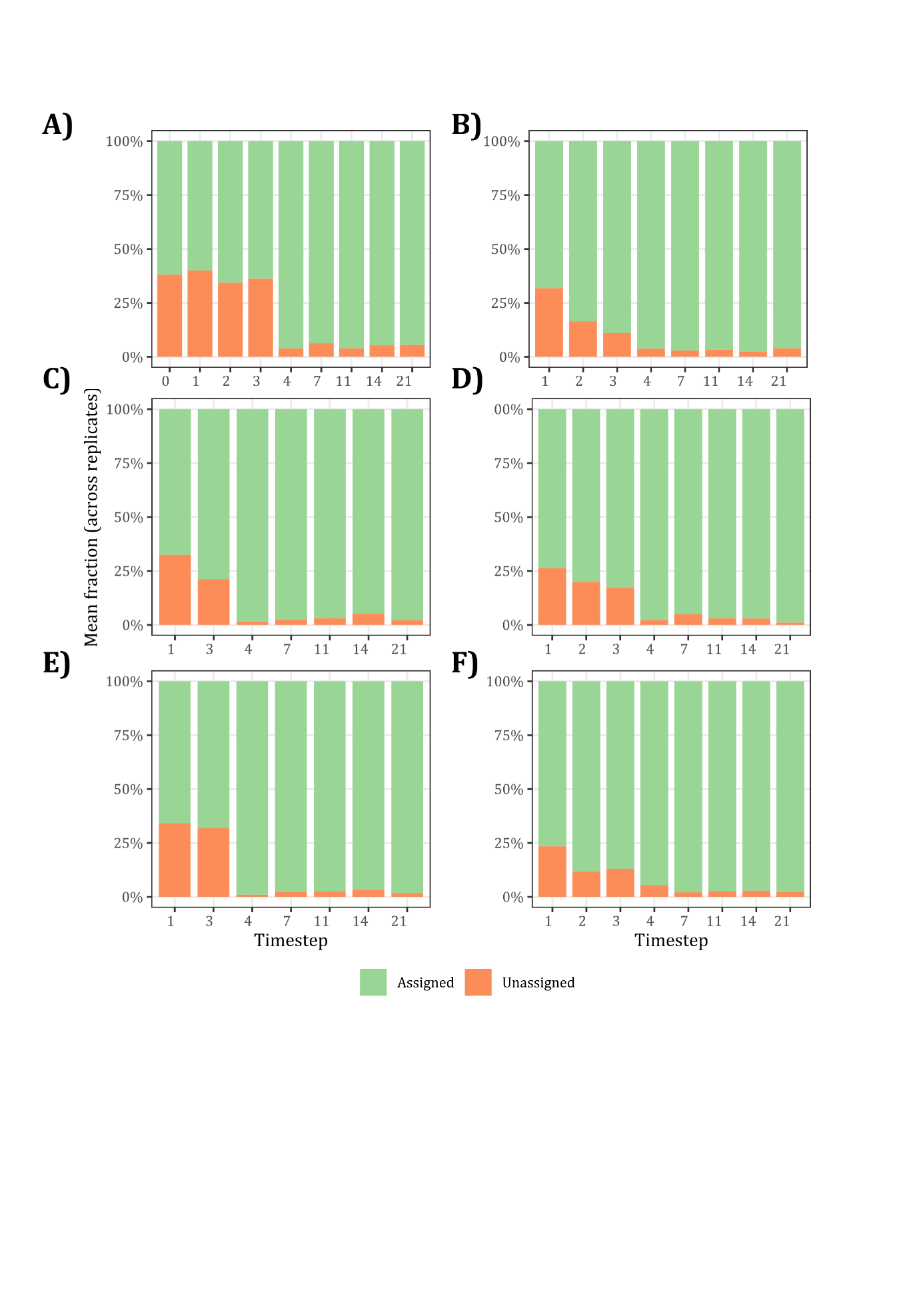


Figure S8: ****Temporal distribution of taxonomically assigned and unassigned reads across treatments.**** Mean fraction of assigned (green) and unassigned (orange) reads across replicates over time. Panels represent (A) native soil, (B) LB, (C) M9, (D) M9 + glucose, (E) M9 + lactose, and (F) R2A-SE medium.
